# Intramolecular Hydrogen Bonding in Thermally Activated Delayed Fluorescence Emitters: Is There Evidence Beyond Reasonable Doubt?

**DOI:** 10.1021/acs.jpclett.2c00907

**Published:** 2022-08-25

**Authors:** Matthias Hempe, Nadzeya A. Kukhta, Andrew Danos, Andrei S. Batsanov, Andrew P. Monkman, Martin R. Bryce

**Affiliations:** † Chemistry Department, Durham University, South Road, Durham DH1 3LE, U.K.; ‡ Materials Science and Engineering Department, University of Washington, Seattle, Washington 98195, United States; § Physics Department, Durham University, South Road, Durham, DH1 3LE, U.K.

## Abstract

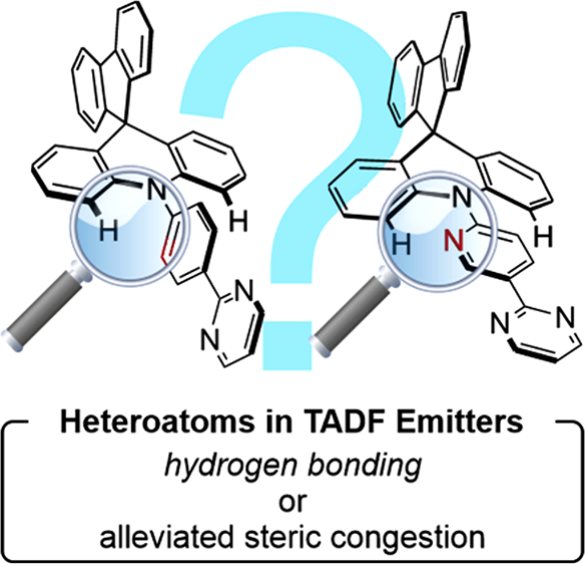

Intramolecular hydrogen bonding between donor and acceptor segments in thermally activated delayed fluorescence (TADF) materials is now frequently employed to—purportedly—rigidify the structure and improve the emission performance of these materials. However, direct evidence for these intramolecular interactions is often lacking or ambiguous, leading to assertions that are largely speculative. Here we investigate a series of TADF-active materials incorporating pyridine, which bestows the potential ability to form intramolecular H-bonding interactions. Despite possible indications of H-bonding from an X-ray analysis, an array of other experimental investigations proved largely inconclusive. Instead, after examining computational potential energy surfaces of the donor–acceptor torsion angle we conclude that the pyridine group primarily alleviates steric congestion in our case, rather than enabling an H-bond interaction as elsewhere assumed. We suggest that many previously reported “H-bonding” TADF materials featuring similar chemical motifs may instead operate similarly and that investigation of potential energy surfaces should become a key feature of future studies.

Thermally activated delayed fluorescence (TADF) in purely organic compounds now frequently underpins high-performance light-emitting applications such as organic light-emitting diodes (OLEDs),^1,2^ sensors,^3^ photocatalysis,^4^ or fluorescence labeling,^5,6^ as the TADF mechanism theoretically allows for the utilization of 100% of excitons for light emission. To enable efficient TADF, spin mixing of singlet and triplet excited states requires energy alignment of states of different orbital characters.^7^ This can be realized in donor–acceptor (D-A) compounds with well-separated frontier molecular orbitals (FMOs), which typically possess near-perpendicular D-A geometries and intramolecular charge-transfer (CT) excited states.^8^ While other classes of materials can also exhibit TADF without requiring perpendicular excited-state geometries—most notably through the multiple-resonance TADF (MR-TADF)^9−36^ and other upper-state crossing^13,14^ mechanisms—for the more commonly reported “vibronically coupled” D-A materials this perpendicularity is understood to be a strong requirement, at least in the excited state. Small energy splitting between excited CT singlet and localized excitonic (LE) triplet states (Δ*E*_ST_) in such materials can be overcome by thermal energy, allowing reverse intersystem crossing (rISC) enabled by a combination of spin–orbit coupling (SOC) and spin-vibronic coupling of these states.^15−21^

Despite their ability to harvest triplet states, CT states with near-perpendicular D-A geometries frequently possess low oscillator strength, resulting in unsuitably low photoluminescence quantum yields (PLQYs) and/or long singlet exciton lifetimes.^22−25^ Hence, efficient TADF molecules must balance these competing factors, with designs that allow for some torsional/vibrational flexibility (a perturbation that enables emission) centered about a near-perpendicular “compromise” average geometry (enabling rISC). Reports in which the D-A geometry is influenced by external groups to understand or control TADF properties are now commonplace.^22,26−43^ Successful strategies can include rigidification of the overall D-A structure or major modification of the average molecular geometry (including D-A angle or axial/equatorial conformers) through molecular design. Incorporation of heteroatoms to induce attractive intramolecular interactions, most commonly N···H hydrogen bonds, is also now frequently reported (see Supporting Information section 2 for selected relevant examples).^37−57,63^

In this latter approach, improved quantum yields, narrower emission spectra, or enhanced SOC compared to control materials are commonly attributed to N···H or similar intramolecular interactions in the heteroatom-substituted material.^38−57^ Although plausibly arising from increased D-A planarization (shifting toward a more optimum “compromise” average geometry) or rigidification of the molecular structure (deactivating some vibrational quenching pathways) direct evidence for intramolecular H-bonding is, to the best of our knowledge, only ever inferred rather than conclusively demonstrated. These inferences are typically based on interatomic distances derived from single-crystal X-ray analysis and/or computational calculations, sometimes in comparison with a non-N-heteroaromatic analogue. The weakness of such inferences is that the ground-state molecular conformation in single crystals can be profoundly modified by intermolecular interactions (i.e., packing forces, typically π–π stacking) and do not necessarily reflect the reality of optoelectronic measurements of excited states in solutions and/or host-dispersed films. Single-point computational comparisons also give limited insight, as while these can identify short interatomic distances, they cannot conclude whether these distances are due to intramolecular attractive forces or arise from other features of molecular design enforcing a particular geometry (e.g., alleviated steric strain). Experimentally, deeper insight is confounded by the impossibility of preparing truly unambiguous “control” materials; incorporating a basic H-bond acceptor unit (e.g., a pyridine compared to a phenyl ring) will also unavoidably change other steric^32^ and electronic properties of the system (e.g., highest/lowest molecular orbital energies, Δ*E*_ST_), any of which can impact the TADF performance to an equal or greater extent than a speculated H-bonding interaction.

To investigate intramolecular H-bonding in TADF materials, we prepared a set of model D-A compounds **1**–**3** featuring pyridine and pyrimidine groups, based on a structure previously reported by Yasuda et al. (see Supporting Information section 3 for details of synthesis and crystallization).^58^ X-ray crystal structures (Figure 1) revealed in each case a near-perpendicular D-A twist (τ) and essentially single intersegmental C–N bond lengths (*a*), indicating that the nitrogen atom at the *ortho* position of the bridging unit does not significantly change the electronic conjugation between the D and A moieties. The acridine moiety is slightly folded along the N···C(spiro) vector, more strongly in compounds **2** & **3** (θ ca. 15° for **1** vs 32–36° for **2** and **3**), forming the central acridine ring into a pseudoboat conformation. This folding was recently investigated for a spiro-fluorene substituted acridine donor^27^ and was also observed in crystals of other acridine-containing TADF emitters.^7,30,59−62^ This increased folding in **2** and **3** could potentially arise from intramolecular hydrogen bonding but also potentially from alleviated D-A steric repulsions as the donor-facing C–H group on the acceptor is replaced by a more compact N atom.

**Figure 1:**
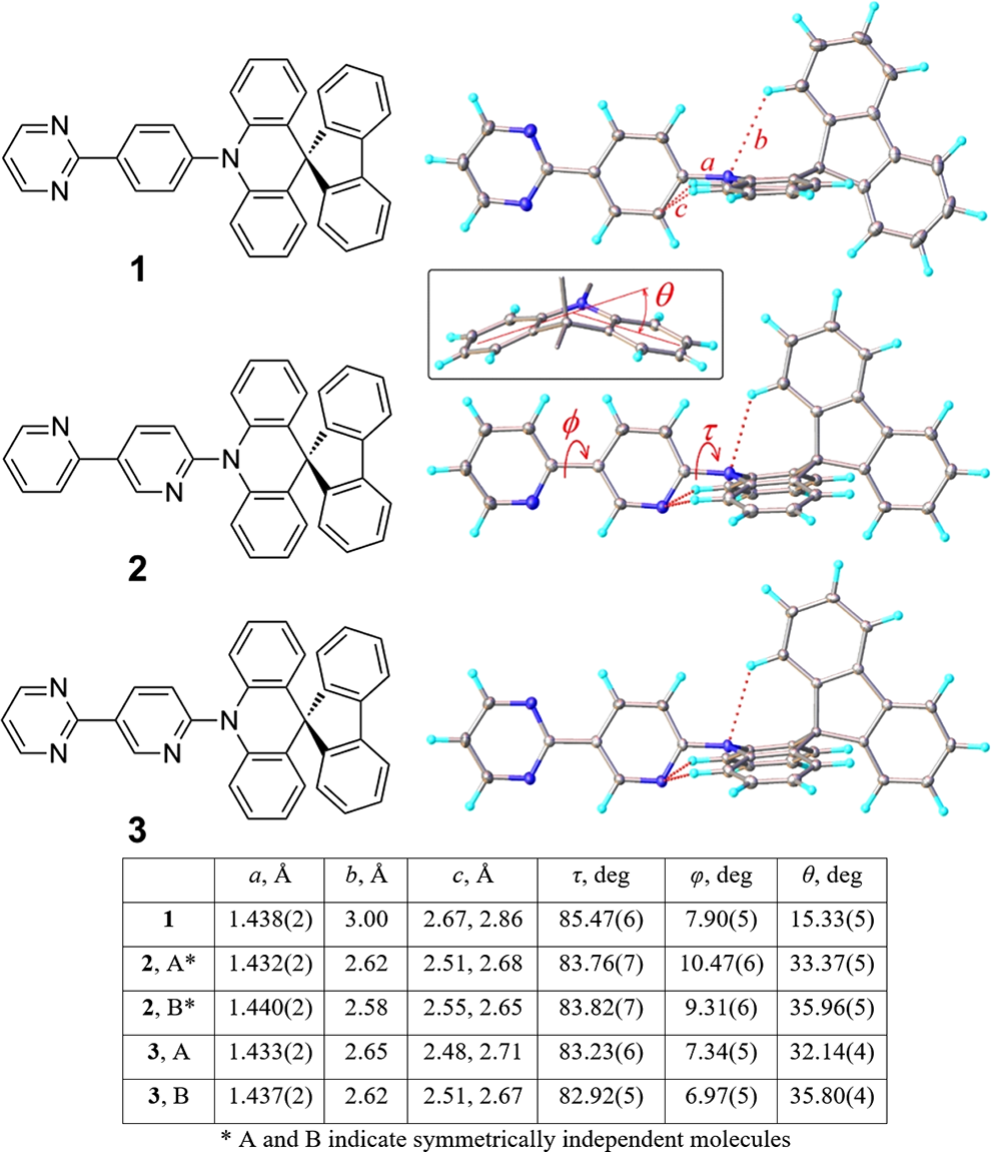
Chemical and X-ray molecular structures of compounds **1**–**3** and relevant geometrical parameters. (inset) Acridine folding.

The shortest intersegmental N···H contacts were ca. 2.5–2.6 Å for **2** and **3**, compared to C···H distances of ca. 2.7–2.9 Å in **1**. While these N···H distances are short enough to potentially result from intramolecular hydrogen bonding, we note that, due to the intersegmental orthogonality, the direction of the sp^2^ nitrogen lone pair does not actually match the direction to the adjacent hydrogen atom of the acridine unit, as would be expected for an H-bond. For other atom pairs with even larger N···H distances, the potential for a structure-determining H-bonding interaction appears even less likely, revealing weakness in the assertions that H-bonding is a structurally significant effect in other reports of similarly structured TADF materials.

In other works, analogous X-ray interatomic distances are sometimes used alone to attribute intramolecular H-bonding as the root cause of differences in TADF performance (examples in Table S1). We note the confounding possibility of intermolecular H-bonding interactions existing alongside other crystal packing forces, which may inextricably influence the observed structural parameters (Figure S23). Furthermore, while TADF is an excited-state process, X-ray structures (and less-expensive calculated structures) can only probe the ground-state geometry. As illustrative examples, in the TADF emitters pDTCz-DPmS^49^ and PXZ–PPO^57^ (entries 7 and 15 in Table S1 in Supporting Information) the D-A units are shown to be nearly coplanar in the ground state. However, further investigation of PXZ–PPO demonstrated that TADF originates from a highly twisted excited state in that material (Figure S3b of that work).^57^ It is therefore inappropriate to rely solely on ground-state or X-ray structures for interpreting the properties of TADF materials, which are rarely measured in crystalline form and which perform their triplet conversion in the excited state.

Here, exhaustive experimental investigations were also performed in the hope to uncover alternative, unambiguous evidence of H-bonding. In ^1^H NMR spectroscopy, intramolecular H-bonding is expected to affect acridine proton signal symmetry and/or result in a severe deshielding of the donor segment proton adjacent to the nitrogen of the acceptor segment bridge in **2** and **3**. Instead, comparing the chemical shifts of the signals in the ^1^H NMR spectra of **1**–**3** (Figure S15), a similar but minor downfield shift of all acridine proton signals of **2** and **3** can be observed. Since this occurs for all acridine signals regardless of their proximity to the nitrogen atom of the bridging heterocycle, it is not reasonable to attribute this to a localized intramolecular H-bonding interaction. Rather, as previously observed for phenothiazine segments in D-A compounds, a comparable downfield shift of all donor proton signals can be observed when comparing planar to slightly folded conformers of the respective donor units.^33^ This downfield shift is therefore in line with reduced electronic conjugation within the donor segment for the more folded structures of **2** and **3** and also follows trends in the cyclic voltammetry data discussed in the Supporting Information (section 9). Alternatively or additionally, a reduction of the intersegmental dihedral angle in **2** and **3** due to alleviated steric hindrance could impact the intersegmental electronic conjugation in solution and result in a minor downfield shift of the acridine proton signals of **2** and **3** as compared to **1**.

To further investigate the structural dynamic differences, compounds **1**–**3** were also probed by variable-temperature ^1^H NMR (Supporting Information, section 5). Down to −80 °C, we observed no signal coalescence/splitting or broadening that might be indicative of intramolecular H-bonding for **1**–**3**.

As TADF materials have predominantly emissive applications, the photophysical properties of compounds **1**–**3** were investigated comprehensively (Figure 2, with additional discussion in Supporting Information section 7). In Zeonex films the steady-state absorption and photoluminescence (PL) bands of the three materials are all different, with the absorbance spectra showing direct CT excitation bands at longer wavelengths (350–400 nm). The PL spectra have predominately broad CT-like emission with some weakly structured emission also evident, reminiscent of what was previously reported for similar acridine-pyrimidine TADF materials.^26^ This hybrid spectral shape arises from mixed CT-LE excited-state character in the nonpolar Zeonex host, which evolves from TADF to room temperature phosphorescence (RTP) in time-resolved measurements (Figure S25, 5 wt % films). This outcome is unsurprising, given the large Δ*E*_ST_ values in the Zeonex host (0.34 eV for **1** and **2**, and 0.16 eV for **3**). The PLQYs of the 1 wt % films were also different, the highest being that for material **3** (55%, as compared to 11% and 24% in **1** and **2**, respectively). The phosphorescence spectra of **1**–**3** collected from 5 wt % films are nearly identical, indicating strongly that this originates from the spiroacridine unit common to all three. Time-resolved emission decays were also collected in dichloromethane (DCM), with the increased polarity of this solvent red-shifting the PL emission, narrowing the Δ*E*_ST_, and tipping the balance from mixed TADF/RTP to pure TADF in all three materials (Figure 2b, contour plots of time-resolved spectra Figure S24). The decays demonstrate the strongest delayed fluorescence (DF) for material **3**; materials **1** and **2** have much weaker DF, although the emission decays more rapidly for **1** than for **2**.

**Figure 2:**
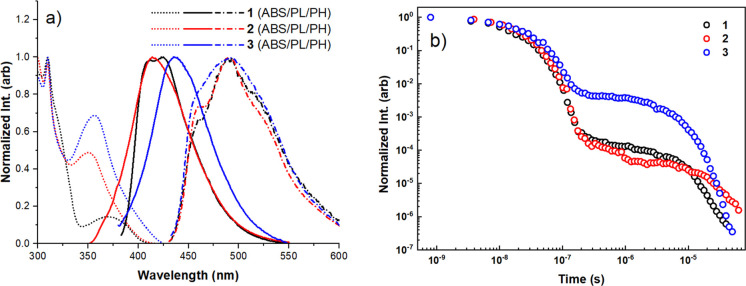
(a) Steady-state absorption (ABS), photoluminescence (PL), and phosphorescence (PH, 80 K after 10 ms delay) of Zeonex films (1 wt % for PL, 5 wt % for ABS and PH). (b) Time-resolved emission decay of degassed DCM solutions (1 mgml^–1^).

While there are significant differences between their optical properties, attributing any of these to a single source is frustrated by the intractable electronic effects of the pyridine nitrogen in materials **2** and **3**. The possibility of intramolecular H-bonding cannot be excluded from these results, but simultaneously the increased acceptor strength associated with the pyrimidine group in **3** is entirely sufficient to explain the red-shifted PL spectrum. This change in electronic state energies leads to reduced Δ*E*_ST_, and the improved TADF performance in time-resolved measurements without needing to additionally invoke an explanation involving intramolecular interactions. Indeed, if intramolecular interactions were the primary cause of these changes, we would expect to see much more similar behavior in **2** as compared to **3** (both with the potential for intramolecular H-bonding), whereas in practice **1** and **2** are much more alike despite the presence of the pyridine site in **2** but not in **1**. In similar studies (Table S1), improved PLQYs akin to what is observed here for **3** are often used to post hoc rationalize the claim of intramolecular H-bonding interactions.

With all available experimental avenues providing at best ambiguous evidence for intramolecular H-bonding in compounds **1**–**3**, we turned to density functional theory (DFT) calculations for additional insight. In other similar reports DFT is often used in a similar capacity as X-ray structures, with short N···H distances used to support the assertion of intramolecular H-bonding.^38,45,47,48,50−54,57,63^ As noted above though, attractive interactions are not the only molecular feature that can cause short N···H interatomic distances, and so their presence in calculations does not immediately confirm an H-bonding interaction.

Investigations of the relaxed S_0_ calculated geometries (rCAM-B3LYP/6-31G(d)) were relatively uninformative. In agreement with the X-ray structures, near-perpendicular D-A dihedral angles were observed in all cases alongside slight acridine folding (22–24°). Investigation of the excited states (TDA-DFT CAM-B3LYP/6-31G(d)) revealed similarly perpendicular structures as well as trends in highest occupied and lowest unoccupied molecular orbitals (HOMO/LUMO, Figure S28), oscillator strengths (Figure S29), and excited-state energy splitting (Figure S31) that are broadly in line with those observed in experimental cyclic voltammetry and optical spectroscopy (Supporting Information sections 9 and 7). Similar to our previous report,^26^ we also observe rocking of the acceptor relative to the plane of the donor (Figure 3, green angles). While this rocking at first appears enforced by N···H attractive forces between the acceptor pyridine and spirofluorene H atoms (thus absent in **1**), we note that the same fluorene distortion occurs in the X-ray structures although with the orientation of the pyridine group reversed (Figure 1). We therefore cannot consider this as conclusive evidence of an H-bonding interaction, as any such attractive interaction would be expected to translate into experimental X-ray structures as well.

**Figure 3:**
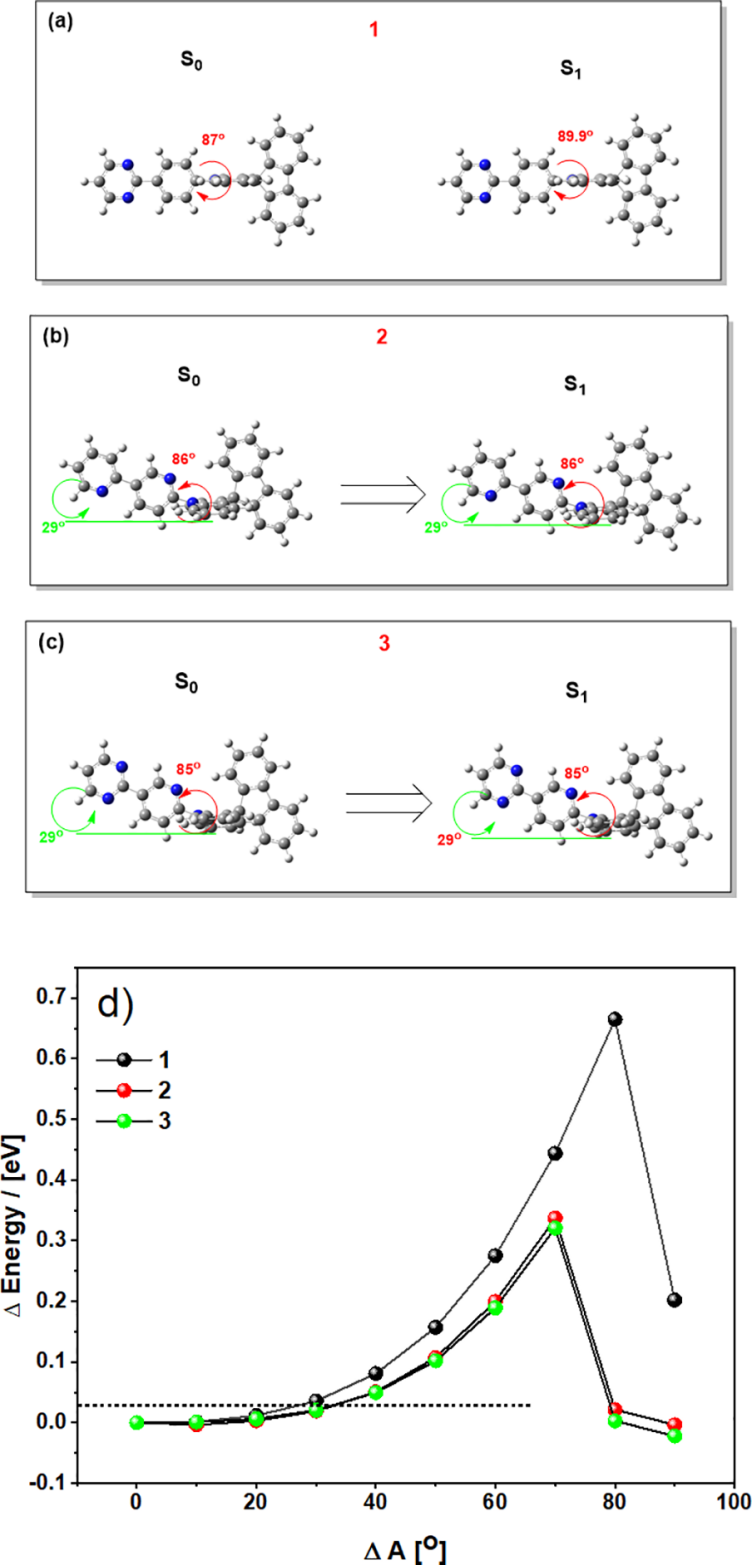
(a–c) Optimized ground-state (rCAM-B3LYP/6-31G(d)) and excited-state (TDA-DFT CAM-B3LYP/6-31G(d)) geometries and (d) PESs of **1**, **2**, and **3** (CAM-B3LYP/6-31G(d)), calculated for D-A bond angular displacement away from the optimized S_1_ geometry. Energies (Δ*E*) and angles (Δ*A)* are plotted relative to their values at the energy-minimized geometry. The dotted line represents 0.025 eV, the approximate value of available thermal energy at room temperature and therefore the extent across the PES these materials are expected to explore.

Examination of natural transition orbitals (NTOs) was also roughly in line with expectations for these CT emitters; the S_0_ → S_1_ NTOs of **1**–**3** feature well-separated hole and particle with minimal overlap, while S_0_ → S_2,3_ excitations revealed additional excited states of increased LE character (Figure S30). The excited-state relaxed D-A dihedral angles vary in the range of 85–90°, which is too small a variance to provide compelling evidence for any excited-state planarization (whether caused by intramolecular H-bonding in **2** and **3**, or otherwise).

Because rISC is a dynamic process involving vibronic coupling of excited states (most commonly through D-A dihedral rocking), a static picture of the relaxed molecular geometry is not always sufficient for deeper understanding.^64^ Consequently, the shape of the potential energy surface (PES) associated with D-A bond rotation was also evaluated (Figure 3d). While some previous studies have investigated similar PESs for D-A motion in TADF materials^44,50,65^ we note that, for ideal comparison, we use only S_1_ excited-state calculations to build our PES. Furthermore, to properly compare the rigidity of the D-A rocking motion between the materials, we also plot our PESs against angular displacement (Δ*A*) away from their individual energy minima, rather than absolute D-A angle. In our case the energy-minima D-A angles are all similar (85–89.9°), and so this consideration makes minimal difference to Figure 3d. In other works though, large differences in relaxed D-A angles between materials (e.g., ∼60° for carbazole, ∼90° for acridine) frustrate direct PES comparison when plotted against absolute D-A angle and absolute (rather than relative) energies at these angles.^44,50,65,66^

Turning to Figure 3d, because intramolecular H-bonding is often invoked as a rigidifying influence on molecular geometry, we expected it to manifest as a steepening of the PES. Instead, the rotational barrier of **1** with a phenylene spacer is steeper than those of **2** and **3** bearing the pyridinyl spacer, the opposite of what would be expected for an attractive or rigidifying intramolecular interaction. Similar results are also observed for calculations using rBMK and rPBE0 computational methods (Figures S33 and S34).

In the absence of conclusive evidence for H-bonding through the previously discussed experimental methods and additionally armed with compelling PES evidence that H-bonding is *not* active in these materials, we are forced to consider alternative explanations. Recently pyrazine-core TADF materials have been reported to enjoy decreased steric congestion, leading to more rotatable carbazole donors and imparting resistance to dimerization.^32^ The weight of evidence points to a similar effect in our case, with the decreased steric congestion in materials **2** and **3** explaining their wider PES curves in Figure 3d as well as their greater extent of acridine folding in Figure 1 (consistently folded toward the pyridine nitrogen). Alleviation of steric crowding is also consistent with the near-perpendicular D-A geometries enjoyed by all three materials, as expanded steric freedom would not directly impact the equilibrium D-A angle.

Applying the same logic more broadly, we suggest that H-bonding may not play as significant a role as previously claimed in other analogous TADF systems (where H-bonding is often assumed or inferred from X-ray data or single-point calculations). While H-bonding may indeed be active in these other systems, as in our case, the differences observed in photophysical properties attributed to H-bonding could instead arise from electronic and/or steric differences induced by the heteroatom-containing substituent. Firmer identification of H-bonding in such systems will likely require deeper investigation of PES curves (plotted against Δ*A*) as in Figure 3d.

In conclusion, we have investigated intramolecular H-bonding in a series of TADF materials bearing phenylene or pyridine bridges. Other reports propose the existence of important H-bonding interactions in similar chemical systems; in our case we find the evidence for H-bonding interactions is inconclusive at best and wholly absent in an appropriate comparison of computational PESs of compounds **1**–**3**. We conclude that alleviation of steric congestion is a significantly more plausible explanation for the differences in material properties we observe here. Similar conclusions may be more widely applicable to previous reports that claim intramolecular H-bonding as a design feature of enhanced TADF materials. We therefore suggest that there remains ample scope for reasonable doubt concerning intramolecular H-bonding in these types of materials.

## Supporting Information Available

Literature overview; synthetic details; ^1^H and ^13^C NMR spectra (including VT NMR data); additional crystallography and computational data; CV traces and data; additional absorption and (time-resolved) emission spectra (PDF)

## Notes

CIF files have been deposited with the Cambridge Structural Database, CCDC 2159816 (compound **1**), 2159817 (compound **2**), and 2159818 (compound **3**).
