# Supplementary material for: Intramolecular Hydrogen Bonding in Thermally Activated Delayed Fluorescence Emitters: Is There Evidence Beyond Reasonable Doubt?
Source: J Phys Chem Lett. 2022 Aug 25;13(35):8221–7. doi: 10.1021/acs.jpclett.2c00907 (PMC9465681; doi:10.1021/acs.jpclett.2c00907)
Supplement: Supplementary file 1 — jz2c00907_si_001.pdf [file jz2c00907_si_001.pdf]

Supporting Information

Intramolecular Hydrogen Bonding in Thermally Activated Delayed Fluorescence Emitters: Is There Evidence Beyond Reasonable Doubt?

Matthias Hempe,[a]† Nadzeya A. Kukhta,[a, b]† Andrew Danos,*[c] Andrei S. Batsanov,[a] Andrew P. Monkman,[c] and Martin R. Bryce*[a]

[a]Chemistry Department, Durham University, South Road, Durham, DH1 3LE, UK
E-Mail: m.r.bryce@durham.ac.uk

[b]Materials Science and Engineering Department, University of Washington, Seattle, Washington 98195, United States

[c]Physics Department, Durham University, South Road, Durham, DH1 3LE, UK
E-Mail: andrew.danos@durham.ac.uk

1.	General experimental details	2
2.	Literature Overview	4
3.1.	Synthesis of 2-bromodiphenylamine 6	8
3.2.	Synthesis of 10*H*-spiro[acridine-9,9'-fluorene] 8	9
3.3.	Synthesis of 2-(4-bromophenyl)pyrimidine 13	10
3.4.	Synthesis of 2-(6-bromopyridin-3-yl)pyrimidine 15	10
3.5.	Synthesis of 6’-bromo-2,3’-bipyridine 14	11
3.6.	Synthesis of Donor-Acceptor compounds 1-3	12
4.	NMR spectra	15
4.1.	2-Bromodiphenylamine 6	15
4.2.	10*H*-Spiro[acridine-9,9‘-fluorene] 8	16
4.3.	2-(4-Bromophenyl)pyrimidine 13	17
4.4.	2-(6-Bromopyridin-3-yl)pyrimidine 15	18
4.5.	6’-Bromo-2,3’-bipyridine 14	19
4.6.	Compound 1	20
4.7.	Compound 2	21
4.8.	Compound 3	22
5.	VT-NMR	24
6.	X-Ray Crystallography	28
7.	Photophysical properties	31
8.	Theoretical calculations	36
9.	Cyclic Voltammetry	46
10.	References	48

1. General experimental details

Solvents and reagents: Solvents were dried using an Innovative Technology solvent purification system and were stored in ampules under argon. Reagents were obtained from commercial sources and were used without further purification. Moisture and/or air sensitive experiments were conducted using thoroughly dried glassware under argon atmosphere.

NMR-Spectra: ^{1}H-NMR spectra were recorded on Bruker AV400, Varian VNMRS 600 and 700 spectrometers operating at 400, 600 and 700 MHz, respectively. 13C-NMR spectra were recorded on the same instruments at 100, 150 and 175 MHz. Chemical shifts (δ) in 1H-NMR and 13C-NMR spectra are reported in ppm and were referenced against the residual solvent signal as reported in the literature.[1] The fine structure of proton signals was specified as s (singlet), d (doublet), t (triplet), q (quartet), m (multiplet), br (broad).

Flash-chromatography was carried out on silica gel 60 (40-63 µm) purchased from Fluorochem.

Mass spectra: High resolution mass spectrometry was carried out on a Waters LCT Premier XE using ASAP ionization with TOF detection. Samples were analyzed directly as solids.

Melting points were carried out on a Stuart SMP40 machine with a ramping rate of 4°C min^{-1}. Videos were replayed manually to determine the melting point or melting range.

X-Ray single-crystal diffraction experiments were performed on a Bruker 3-circle D8 Venture diffractometer with a PHOTON 100 CMOS area detector, using Mo Kα radiation (λ = 0.71073 Å) from an Incoatec IµS microsource with focusing mirrors. Crystals were cooled to 120 K using a Cryostream (Oxford Cryosystems) open-flow N_2 gas cryostat. The data were processed using APEX3 v.2016.1-0, reflection intensities integrated using SAINT v8.38A software (Bruker AXS, 2016) and scaled using SADABS-2016/2 program.[2] The structures were solved by dual-space intrinsic phasing method using SHELXT 2018/2 program[3] and refined by full-matrix least squares using SHELXL 2018/3 software[4] on the OLEX2 platform.[5]

Cyclic voltammetry was conducted using an electrochemical cell comprised of platinum electrode with a 1 mm diameter of working area as a working electrode, an Ag/AgCl electrode as a reference electrode and a platinum coil as an auxiliary electrode. Cyclic voltammetry measurements were conducted at room temperature at a potential rate of 50 mV s^{-1} and were calibrated against the internal ferrocene/ferrocenium redox couple. The measurements were conducted in ca. 1.0 mM concentrations of all compounds in 0.1 M solutions of $TBAPF_6$ in *N*,*N*-dimethylformamide (abs.).

Photophysical Characterization. Steady-state absorption and emission spectra were measured using a double beam Shimadzu UV-3600 UV/VIS/NIR spectrophotometer and a Horiba Jobin Yvon Fluorolog-3 spectrofluorometer. Time-resolved measurements were performed using a spectrograph

and a Stanford Computer Optics 4 Picos ICCD camera, where samples were excited with a Nd:YAG laser (EKSPLA), 10 Hz, 355 nm. PLQYs were measured using a calibrated Quanta-φ integrating sphere with coupled Jobin Yvon FluoroLog-3 spectrometer with PMT detector (0.5 s integration time) and analyzed using FluorEsscence software. The sphere was flushed with N_2 for 30 min prior to measurement to prevent triplet quenching by atmospheric oxygen.

2. Literature Overview

Table S1. Representative structures as overview on molecular design strategies involving potential intramolecular C-H···N interactions (highlighted in red, and inferred where not specified in original references) in light-emitting materials. It should be noted that these molecules will adopt twisted, and even near-perpendicular, conformations between the donor and acceptor subunits in the excited state, which underpins their TADF properties.

#	Structures	Year	Ref	Interaction
1	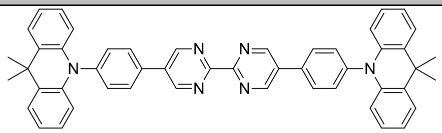 22bpmAc<br>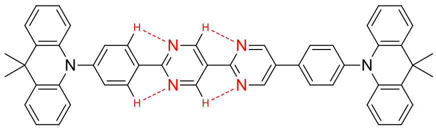 25bpmAc<br>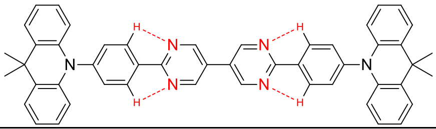 55bpmAc	**2018**	6	Intrasegmental
2	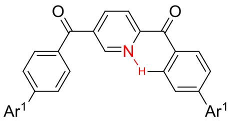 Ar^1 = A (25DAcBPy)<br>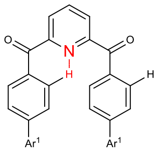 Ar^1 = A (26DAcBPy)<br>Ar^1 = B (26DPXZBPy)<br>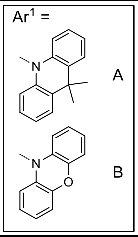	**2019**	7	Intrasegmental
3	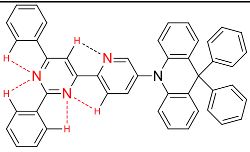 DPAc-4PyPM<br>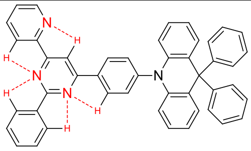 DPAc-6PyPM<br>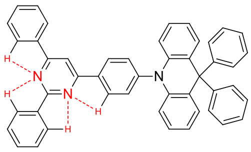 DPAc-TPPM	**2018**	8	Intrasegmental
4	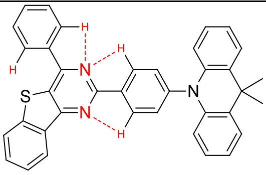 BTPAc<br>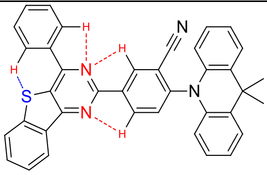 BTPCNAc	**2019**	9	Intrasegmental
5	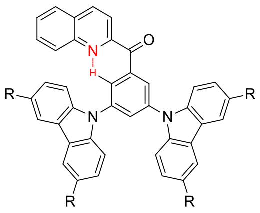 R = H (2QPM-mDC)<br>R = *t*-Bu (2QPM-mDTC)<br>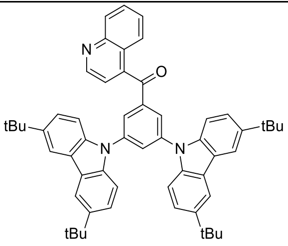 4QPM-mDTC	**2019**	10	Intrasegmental

6	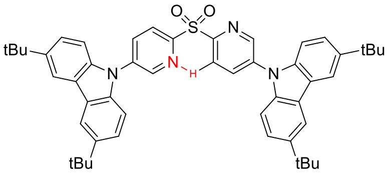<br>pDTCu-2DPyS<br>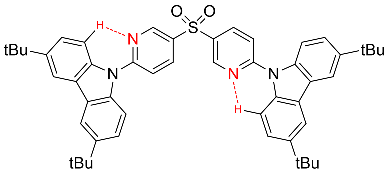<br>pDTCz-3DPyS<br>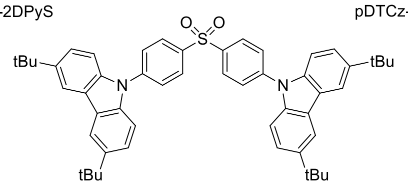<br>pDTCz-DPS<br>(*J. Am. Chem. Soc.* **2012**, *134*, 14706.)	**2019**	11	Intra- and intersegmental
7	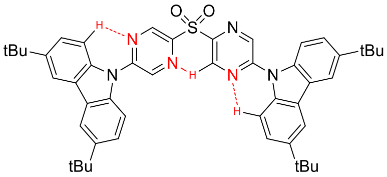<br>pDTCz-DPzS<br>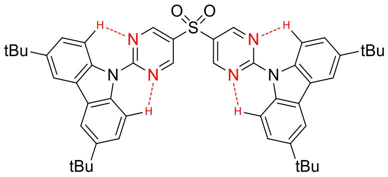<br>pDTCz-DPmS	**2019**	12	Intra- and intersegmental
8	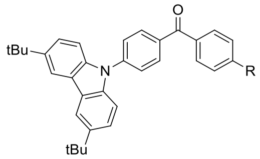<br>R = A (CBM-DMAC)<br>R = B (CBM-PTZ)<br>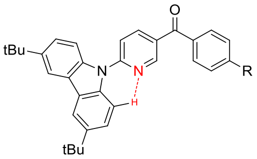<br>R = A (3CPyM-DMAC)<br>R = B (3CPyM-PXZ)<br>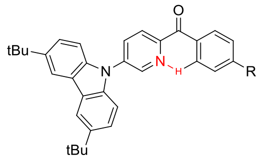<br>R = A (2CPyM-DMAC)<br>R = B (2CPyM-PXZ)<br>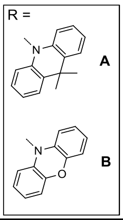	**2020**	13	Intra- and intersegmental
9	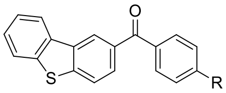<br>MCZ-B-DTM<br>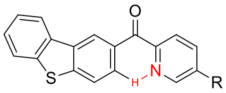<br>MCZ-P2-DTM<br>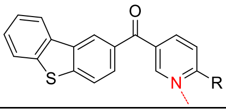<br>MCZ-P3-DTM<br>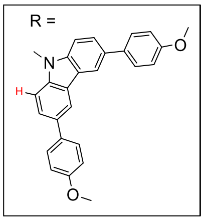	**2019**	14	Intra- and intersegmental
10	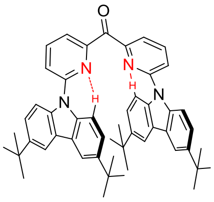<br>2DPyM-*m*DTC<br>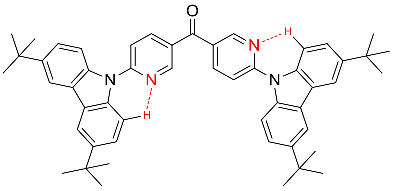<br>3DPyM-*p*DTC	**2017**	15	Intersegmental

11	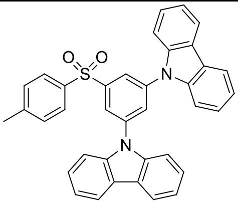 SO-Cz 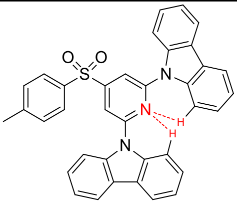 SON-Cz 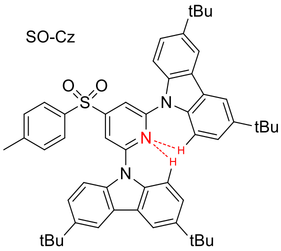 SON-*t*BuCz 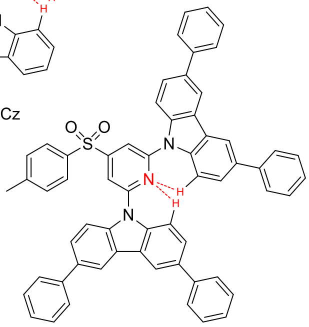 SON-PhCz	**2021**	16	Intersegmental
12	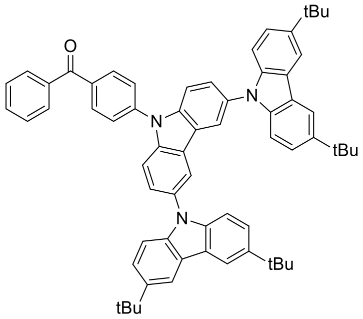 BP-CDTC 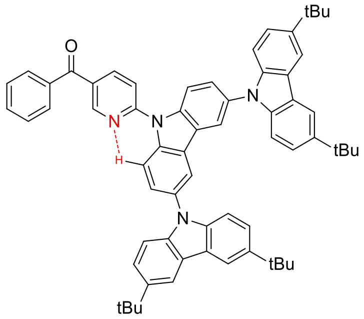 BPy3-CDTC 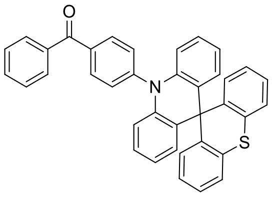 BP-TXDMAc 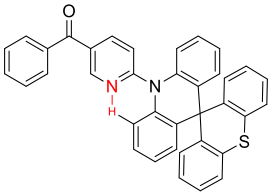 BPy3-TXDMAc	**2019**	17	Intersegmental
13	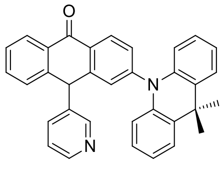 PDAD-DMAC 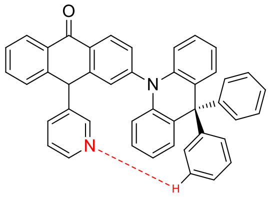 PDAD-DPAC 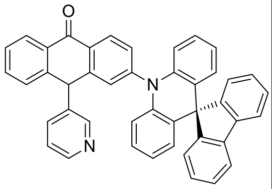 PDAD-SFAC	**2021**	18	Intersegmental
14	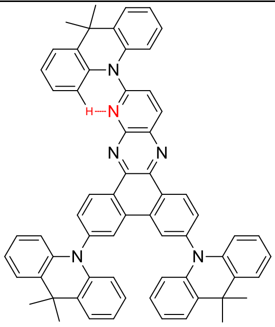 3,6,11-triAC-BPQ 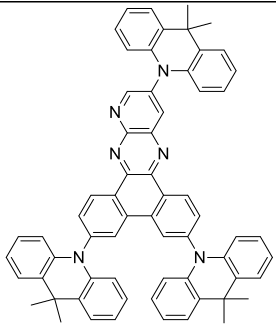 3,6,12-triAC-BPQ	**2020**	19	Intersegmental

15	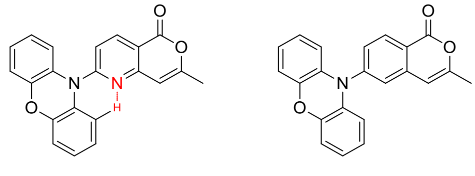 PXZ-PPO PXZ-BOO	**2020**	20	Intersegmental

3. Experimental procedures and characterization data

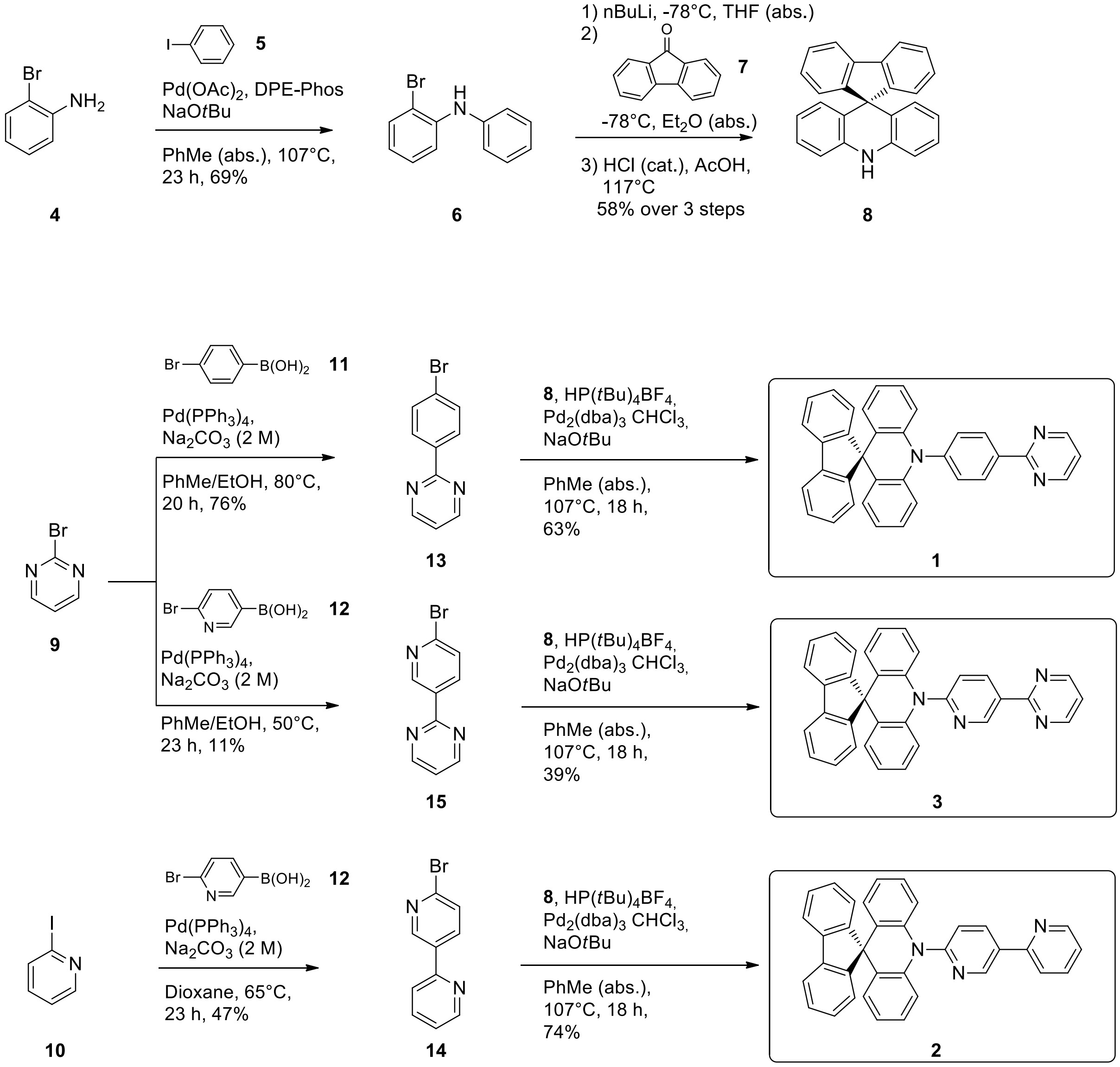

Scheme S1. Overview of the synthetic approach.

The syntheses of the materials **1-3** is based on a modular approach utilizing the donor **8** and respective acceptor bromides **13-15** via Buchwald-Hartwig amination reactions (Scheme S1). The donor segment **8** was synthesized in two steps. Starting with 2-bromoaniline **4**, the diphenylamine compound **6** was obtained in a selective Buchwald-Hartwig cross-coupling reaction using iodobenzene **5** and the bis-dentate ligand DPE-Phos. Afterwards, compound **6** was used in a metalation and subsequent

nucleophilic attack reaction sequence. The intermediate carbinole was not isolated, but subsequently cyclized to obtain the spiro-compound **8**.

The synthesis of the acceptor segments **13** and **15** was based on Suzuki cross-coupling reactions using 2-bromopyrimidine **9** and the respective boronic acids **11** and **12**. Accordingly, 6'-bromo-2,3'-bipyridine **14** was obtained using 2-iodopyridine **10** and the boronic acid **12**.

The synthesis of target compounds **1-3** was achieved by the Buchwald-Hartwig amination reaction using the respective acceptor bromides **13-15** and the acridine compound **8**.

3.1. Synthesis of 2-bromodiphenylamine 6

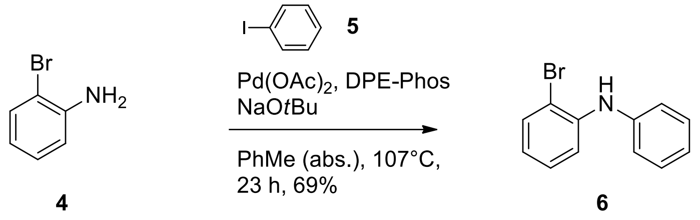

Adapting to a literature procedure[21] and under an argon atmosphere, 10.00 g (58.13 mmol) 2-bromo aniline **4** and 1.88g (0.06 eq., 3.49 mmol) DPE-Phos were dissolved in 60 mL toluene (abs.). To this solution, 11.86 g (1.00 eq., 58.13 mmol) iodobenzene **5** and 392 mg (0.03 eq., 1.74 mmol) $Pd(OAc)_2$ were added. The mixture was heated to 110°C and 6.14 g (1.10 eq., 63.94 mmol) NaO*t*Bu were added. The reaction mixture was stirred at 110°C for 23 h and was subsequently cooled to room temperature. The mixture was filtered through plugs of celite and silica and was concentrated under reduced pressure. The residue was purified by column chromatography on silica (PE/DE, 10:1), yielding product **6** (9.90 g, 69%, 39.90 mmol) as an orange-colored oil, which tends to crystallize after a while.

The analytical data match those reported in the literature.[22]

^{1}H-NMR (CD_2Cl_2, 400 MHz, 294 K) δ (ppm) = 7.54 (d, J = 7.99 Hz, 1.47 Hz, 1H), 7.32 (t, J = 7.91 Hz, 2H), 7.25 (d, J = 8.22 Hz, 1.58 Hz, 1H), 7.21-7.13 (m, 3H), 7.04 (t, J = 7.38 Hz, 1H), 6.75 (m, 1H), 6.12 (s, 1H).

M.p. 118-120°C

3.2. Synthesis of 10*H*-spiro[acridine-9,9'-fluorene] 8

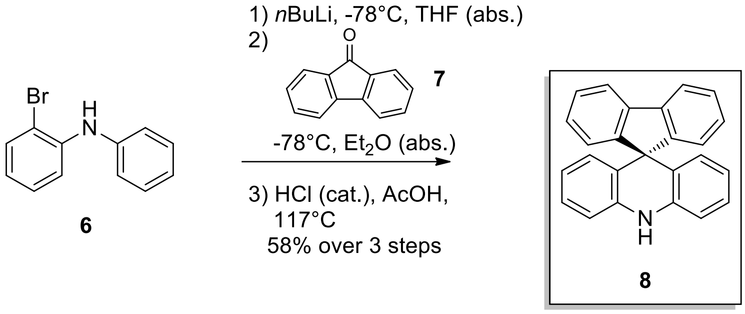

Under an argon atmosphere, 5.40 g (21.76 mmol) 2-bromo-diphenylamine **6** were dissolved in 45 mL THF (dry) and cooled to -78°C. In a separate flask, 21 mL (2 M, 52.45 mmol) *n*-butyl lithium solution in hexane were cooled to -78°C. The precooled solution of the diphenylamine was slowly added to the solution of the n-butyl lithium, resulting in a yellow mixture. The mixture was stirred for 2 h, and a precooled solution of 3.92 g (1 eq., 21.76 mmol) fluorenone **7** in 45 mL Et_2O (dry) was slowly added. The mixture was stirred at -78°C for 5 h and subsequently was quenched by the addition of 20 mL sat. NaCl solution (aq.). The phases were separated and the organic layer was dried over $MgSO_4$ and concentrated, resulting in a red oil. The oil was dissolved in 200 mL glacial acetic acid and was heated to 90°C, after which HCl (conc.) were added. The mixture was heated to 117°C for 8 h and was cooled to room temperature and concentrated *in vacuo*, afterwards. The residue was mixed with 200 mL DCM and was washed with with water (3x 250 mL), sat. $NaHCO_3$ solution (aq., 3x 150 mL) and 200 mL sat. NaCl solution (aq.). The organic layer was dried using $MgSO_4$ and was concentrated under reduced pressure. The resulting residue was purified by column chromatography on silica (*n*Hex/DCM, v/v, 3:1), resulting in an off-white solid. The product was further purified by trituration using hot *n*Hex, resulting in **8** (4.18 g, 58%, 12.61 mmol) as a colorless solid.

The analytical data match those reported in the literature.[23]

^{1}H-NMR (CD_2Cl_2, 400 MHz, 294 K) δ (ppm) = 7.81 (d, J = 7.60 Hz, 2H), 7.37 (t, J = 7.42 Hz, 2H), 7.28 (d, J = 7.60 Hz, 2H), 7.22 (m, 2H), 7.09 (m, 2H), 6.88 (d, J = 8.02 Hz, 1.12 Hz, 2H), 6.57 (t, J = 7.54 Hz, 2H), 6.50 (s, 1H), 6.30 (d, J = 7.80 Hz, 2H).

3.3. Synthesis of 2-(4-bromophenyl)pyrimidine 13

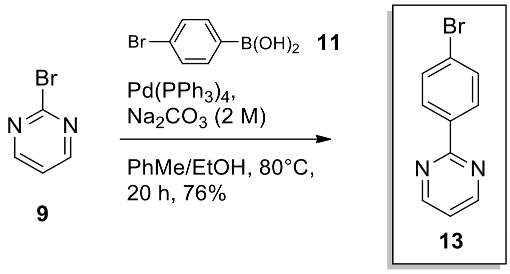

In adaption of a literature procedure,[24] 801 mg (1.5 eq., 5.04 mmol) 2-bromopyrimidine **9** were dissolved in 6 mL toluene and a solution of 1.51 g (3.25 eq., 10.92 mmol) sodium carbonate in 6 mL water was added. The mixture was purged with argon for 20 minutes, after which 116.5 mg (0.03 eq., 0.10 mmol) $Pd(PPh_3)_4$ were added. The mixture was heated to 50°C and a degassed solution of 675 mg (1.00 eq., 3.36 mmol) 4-bromophenylboronic acid **11** in 6 mL ethanol was added dropwise over the period of 2 h. The mixture was stirred for further 18 h at 50°C and subsequently was diluted using 20 mL dichloromethane. The mixture was filtered through plugs of celite and silica, and afterwards was concentrated under reduced pressure. The residue was purified by column chromatography on silica (DCM/Hex, v/v, gradient 1:1→3:1), resulting in product **13** (601 mg, 76%, 2.56 mmol) as an off-white solid.

^{1}H-NMR (CD_2Cl_2, 700 MHz, 298 K) δ (ppm) = 8.79 (d, J = 4.81 Hz, 2H), 8.35 (d, J = 8.63 Hz, 2H), 7.63 (d, J = 8.63 Hz, 2H), 7.23 (t, J = 4.81 Hz, 1H).

^{13}C-NMR (CD_2Cl_2, 175 MHz, 298 K) δ (ppm) = 163.57, 157.27, 136.73, 131.65, 129.65, 125.25, 119.43.

HRMS-ASAP-TOF$^+$ *m/z* calculated for $C_{10}H_8BrN_2$ $[M+H]^+$ 234.9871, found $[M+H]^+$ 234.9866.

M.p. 136-137°C

3.4. Synthesis of 2-(6-bromopyridin-3-yl)pyrimidine 15

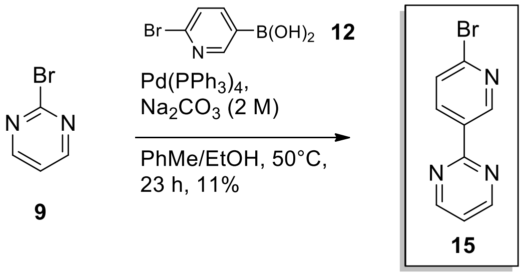

In adaption of a literature procedure,[24] 1.06 g (1.5 eq., 6.69 mmol) 2-bromopyrimidine **9** were dissolved in 7 mL toluene and a solution of 2.00 g (3.25 eq., 14.49 mmol) sodium carbonate in 7 mL water was added. The mixture was purged with argon for 20 minutes, after which 129 mg (0.03 eq., 0.11 mmol)

$Pd(PPh_3)_4$ were added. The mixture was heated to 50°C and a degassed solution of 900 mg (1.00 eq., 4.46 mmol) 2-bromopyridine-5-boronic acid **12** in 7 mL ethanol was added dropwise over the period of 2 h. The mixture was stirred for further 21 h at 50°C and subsequently was diluted using 20 mL dichloromethane. The mixture was filtered through plugs of celite and silica, and afterwards was concentrated under reduced pressure. The residue was purified by column chromatography on silica (*n*Hex/ethyl acetate, v/v, gradient 3:1→2:1), and subsequently was recrystallized from hot ethanol. The product **15** (117 mg, 11%, 0.50 mmol) was obtained as colorless needles.

^{1}H-NMR (CD_2Cl_2, 700 MHz, 298 K) δ (ppm) = 9.36 (s, 1H), 8.83 (d, J = 4.84 Hz, 2H), 8.57 (d, J = 8.28, 2.43 Hz, 1H), 7.61 (d, J = 8.30 Hz, 1H), 7.29 (t, J = 4.84 Hz, 1H).

^{13}C-NMR (CD_2Cl_2, 175 MHz, 298 K) δ (ppm) = 162.06, 157.43, 150.14, 144.23, 137.73, 132.64, 127.86, 120.06.

HRMS-ASAP-TOF$^+$ *m/z* calculated for $C_9H_7BrN_3$ $[M+H]^+$ 235.9823, found $[M+H]^+$ 235.9819.

M.p. 104°C

3.5. Synthesis of 6’-bromo-2,3’-bipyridine 14

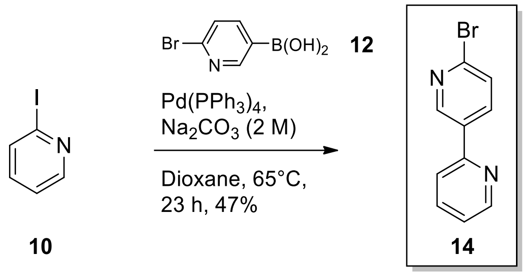

In modification of a literature procedure[25], 1.75 g (1.5 eq., 8.55 mmol) 2-iodopyridine **10** were dissolved in 7 mL dioxane and a solution of 1.97 g (2.5 eq., 14.24 mmol) sodium carbonate in 7 mL water was added. The mixture was purged with argon for 20 minutes, after which 198 mg (0.03 eq., 0.17 mmol) $Pd(PPh_3)_4$ were added. The mixture was heated to 65°C and a degassed solution of 1.15 g (1.00 eq., 5.70 mmol) 2-bromopyridin-5-boronic acid **12** in 7 mL dioxane was added dropwise over the period of 30 min. The mixture was stirred for 24 h at 65°C and subsequently was diluted using 20 mL dichloromethane. The mixture was filtered through plugs of celite and silica, and afterwards was concentrated under reduced pressure. The residue was purified by column chromatography on silica (Hex/ethyl acetate, v/v, 3:1), yielding **14** (632 mg, 47%, 2.69 mmol) as a colorless solid.

^{1}H-NMR (CD_2Cl_2, 700 MHz, 298 K) δ (ppm) = 8.96 (s, 1H), 8.70 (d, J = 4.76 Hz, 1H), 8.22 (d, J = 8.30, 2.60 Hz, 1H), 7.82 (t, J = 1.81 Hz, 1H), 7.78 (d, J = 7.96 Hz, 1H), 7.59 (d, J = 8.30 Hz, 1H), 7.32 (t, J = 4.76 Hz, 1.10 Hz, 1H).

^{13}C-NMR (CD_2Cl_2, 175 MHz, 298 K) δ (ppm) = 153.47, 150.05, 148.44, 142.38, 137.02, 136.71, 134.20, 127.96, 123.14, 120.27.

HRMS-ASAP-TOF$^+$ *m/z* calculated for $C_{10}H_8BrN_2$ $[M+H]^+$ 234.9871, found $[M+H]^+$ 234.9860.

M.p. 90°C

3.6. Synthesis of Donor-Acceptor compounds 1-3

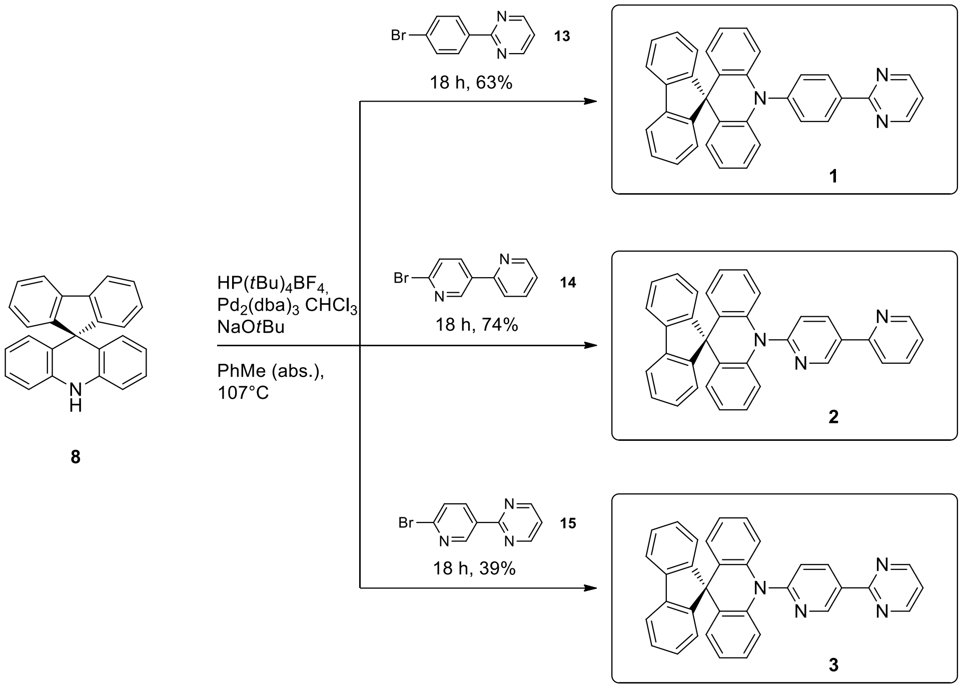

General procedure

Under an argon atmosphere, 1 eq. of the bromide **13-15** and 1.05 eq. of the acridine component **8** were dissolved in toluene (0.1 M, abs., 3.6 mL). To this solution, 0.1 eq. HP(*t*Bu)$_4$BF$_3$ were added and the mixture was purged with argon for 30 min. Afterwards, 0.04 eq. $Pd_2(dba)_3 \cdot CHCl_3$ were added and the mixture was heated to 107°C. At this temperature, 2.5 eq. NaO*t*Bu were added and the mixture was stirred until completion of the reaction. The mixture was cooled to room temperature and was filtered through plugs of celite and silica using DCM as eluent. The solvent was removed under reduced pressure and the residue was purified by column chromatography on silica (DCM/Hex, v/v, gradient 1:1→3:1). After removal of the solvent *in vacuo*, the remaining solid was thoroughly washed using cold *n*-pentane and was dried under high vacuum.

3.6.1. Synthesis of 1

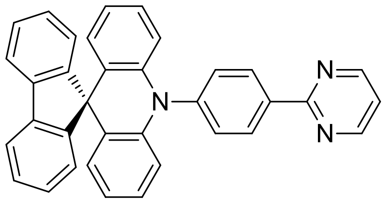

1

Following the general procedure, 84 mg (0.36 mmol) 2-(4-bromophenyl)pyrimidine **13** and 124 mg (1.05 eq., 0.38 mmol) acridine **8** were used to obtain **1** (110 mg, 63%, 0.23 mmol) as a colorless solid.

^{1}H-NMR (CD_2Cl_2, 600 MHz, 298 K) δ (ppm) = 8.90 (d, J = 4.86 Hz, 2H), 8.82 (d, J = 8.57 Hz, 2H), 7.86 (d, J = 7.54, 2H), 7.65 (d, J = 8.57 Hz, 2H), 7.44 (d, J = 7.52 Hz, 2H), 7.42 (t, 2H), 7.27-7.34 (m, 3H), 6.94 (t, J = 7.76 Hz, 1.57 Hz, 2H), 6.58 (t, J = 7.46 Hz, 1.14 Hz, 2H), 6.47 (d, J = 8.46 Hz, 0.88 Hz, 2H), 6.39 (d, J = 7.82 Hz, 1.48 Hz, 2H).

^{13}C-NMR (CD_2Cl_2, 150 MHz, 298 K) δ (ppm) = 163.81, 156.41, 156.49, 143.23, 141.13, 139.19, 137.93, 131.29, 130.84, 128.32, 127.66, 127.44, 127.24, 125.49, 124.77, 120.50, 120.00, 119.50, 114.77, 56.76.

HRMS-ASAP-TOF^{+} *m/z* calculated for $C_{35}H_{24}N_3$ $[M+H]^+$ 486.1970, found $[M+H]^+$ 486.1956.

M.p. 348°C

Crystals for X-ray analysis were obtained by dissolution of the compound in DCM. The solution was layered with *n*-hexane and was subsequent left for slow evaporation of the solvent at room temperature.

3.6.2. Synthesis of 2

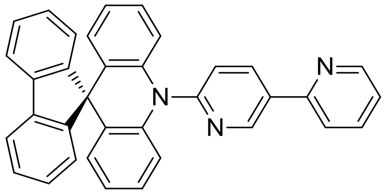

2

Following the general procedure, 86 mg (0.37 mmol) 6’-bromo-2,3’-bipyridine **14** and 130 mg (1.07 eq., 0.39 mmol) acridine **8** gave **2** (131 mg, 74%, 0.27 mmol) as an off-white solid.

^{1}H-NMR (CD_2Cl_2, 700 MHz, 298 K) δ (ppm) = 9.48 (s, 1H), 8.797 (d, 1H), 8.710 (d, J = 8.07 Hz, 2.52 Hz, 1H), 7.95 (d, 1H), 7.90 (t, 1H), 7.86 (d, 2H), 7.69 (d, J = 8.07 Hz, 1H), 7.46 (d, J = 7.53 Hz, 2H),

7.42 (t, 2H), 7.39 (t, 1H), 7.30 (t, 2H), 6.98 (t, 2H), 6.64 (t, 2H), 6.56 (d, *J* = 8.40 Hz, 0.96 Hz, 2H), 6.43 (d, *J* = 7.88 Hz, 1.48 Hz, 2H).

^{13}C-NMR (CD_2Cl_2, 175 MHz, 298 K) δ (ppm) = 155.79, 154.83, 153.93, 150.17, 149.67, 140.52, 139.31, 138.13, 137.08, 134.49, 128.33, 127.70, 127.37, 127.21, 125.88, 125.56, 124.88, 123.11, 121.19, 120.62, 120.00, 115.23, 56.87.

HRMS-ASAP-TOF$^+$ *m/z* calculated for $C_{35}H_{24}N_3$ $[M+H]^+$ 486.1970, found $[M+H]^+$ 486.1971.

M.p. decomp. 280°C

Crystals for X-ray analysis were obtained by dissolution of the compound in DCM-d_2 and subsequent slow evaporation of the solvent at room temperature.

3.6.3. Synthesis of 3

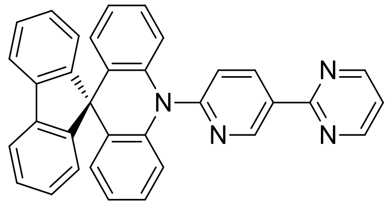

3

Following the general procedure, 86 mg (0.36 mmol) 2-(6-bromopyridin-3-yl)pyrimidine **15** and 127 mg (1.07 eq., 0.38 mmol) acridine **8** gave **3** (69 mg, 39%, 0.14 mmol) as a colorless solid.

^{1}H-NMR (CD_2Cl_2, 700 MHz, 298 K) δ (ppm) = 9.87 (s, *J* = 2.38 Hz, 0.60 Hz, 1H), 9.04 (d, *J* = 8.14 Hz, 2.40 Hz, 1H), 8.92 (d, *J* = 4.87 Hz, 2H), 7.85 (d, *J* = 7.59 Hz, 2H), 7.69 (d, *J* = 8.14 Hz, 0.57 Hz, 1H), 7.44 (d, *J* = 7.53 Hz, 2H), 7.42 (t, *J* = 7.53 Hz, 1.03 Hz, 2H), 7.34 (t, 1H), 7.29 (t, *J* = 7.43 Hz, 1.03 Hz, 2H), 7.00 (t, 2H), 6.66 (t, 2H), 6.65 (d, 2H), 6.44 (d, 2H).

^{13}C-NMR (CD_2Cl_2, 175 MHz, 298 K) δ (ppm) = 162.39, 157.54, 156.36, 155.54, 151.21, 140.43, 139.37, 139.16, 132.56, 128.32, 127.71, 127.33, 127.19, 126.44, 125.55, 124.10, 121.41, 120.01, 119.97, 115.72, 56.95.

HRMS-ASAP-TOF$^+$ *m/z* calculated for $C_{34}H_{23}N_4$ $[M+H]^+$ 487.1923, found $[M+H]^+$ 487.1916.

M.p. 308°C

Crystals for X-ray analysis were obtained by dissolution of the compound in DCM-d_2 and subsequent slow evaporation of the solvent at room temperature.

4. NMR spectra

4.1. 2-Bromodiphenylamine 6

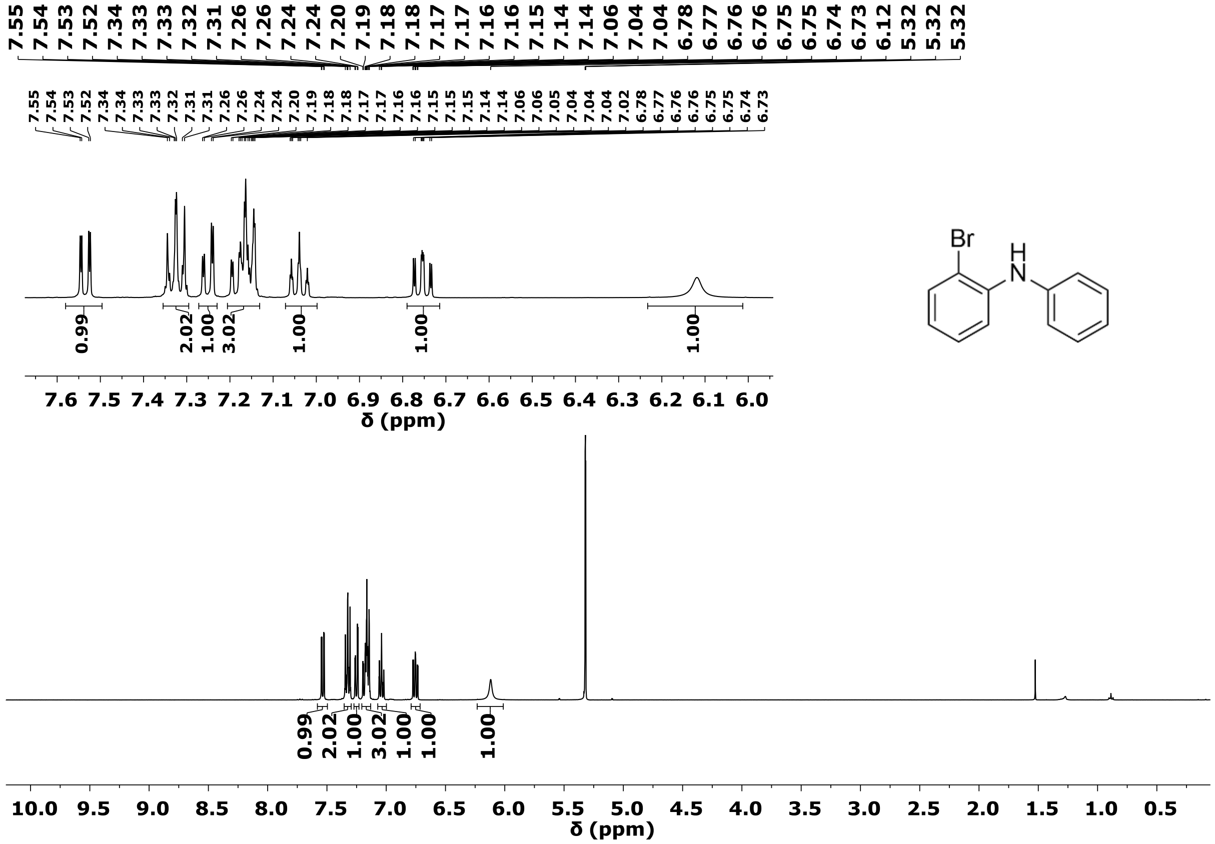

Figure S1. ^{1}H-NMR spectrum of **6** (CD_2Cl_2, 400 MHz, 294 K).

4.2. 10*H*-Spiro[acridine-9,9'-fluorene] 8

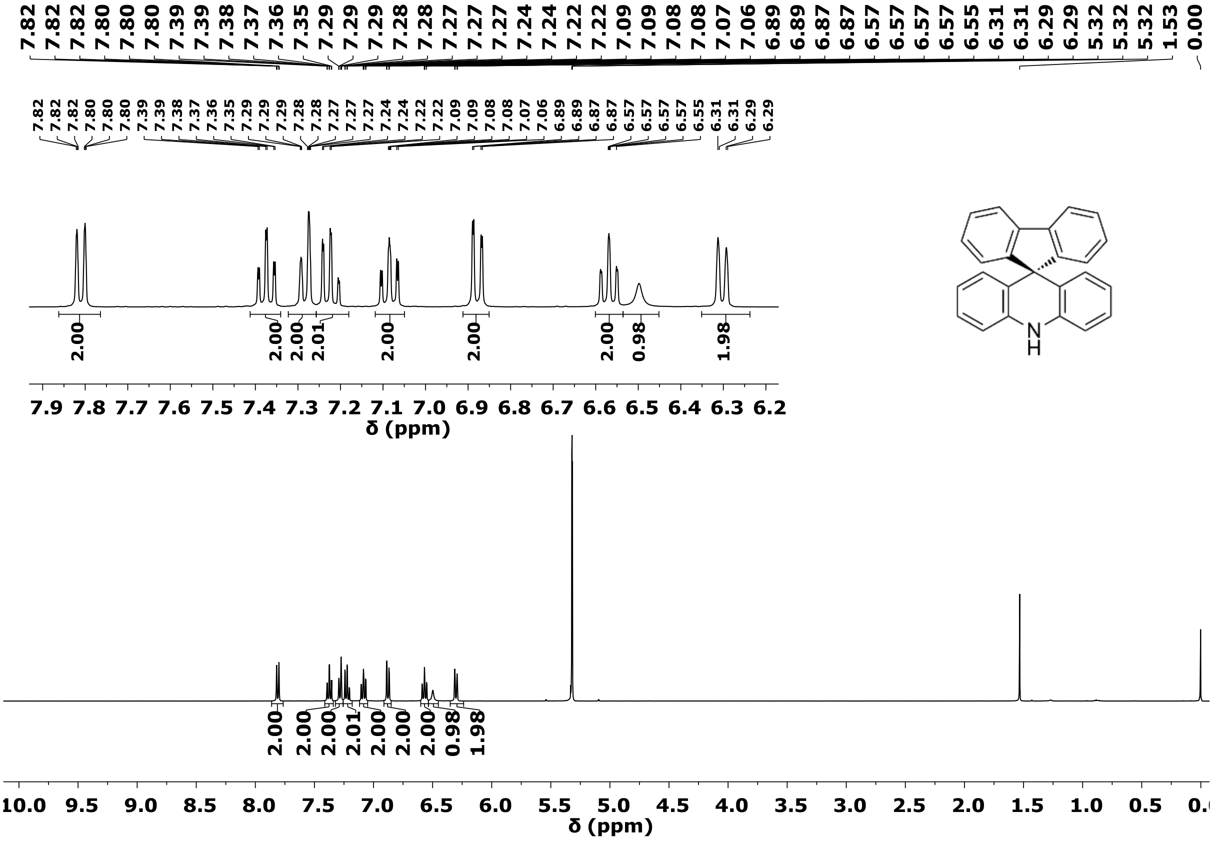

Figure S2. ^{1}H-NMR spectrum of **8** (CD_2Cl_2, 400 MHz, 294 K).

4.3. 2-(4-Bromophenyl)pyrimidine 13

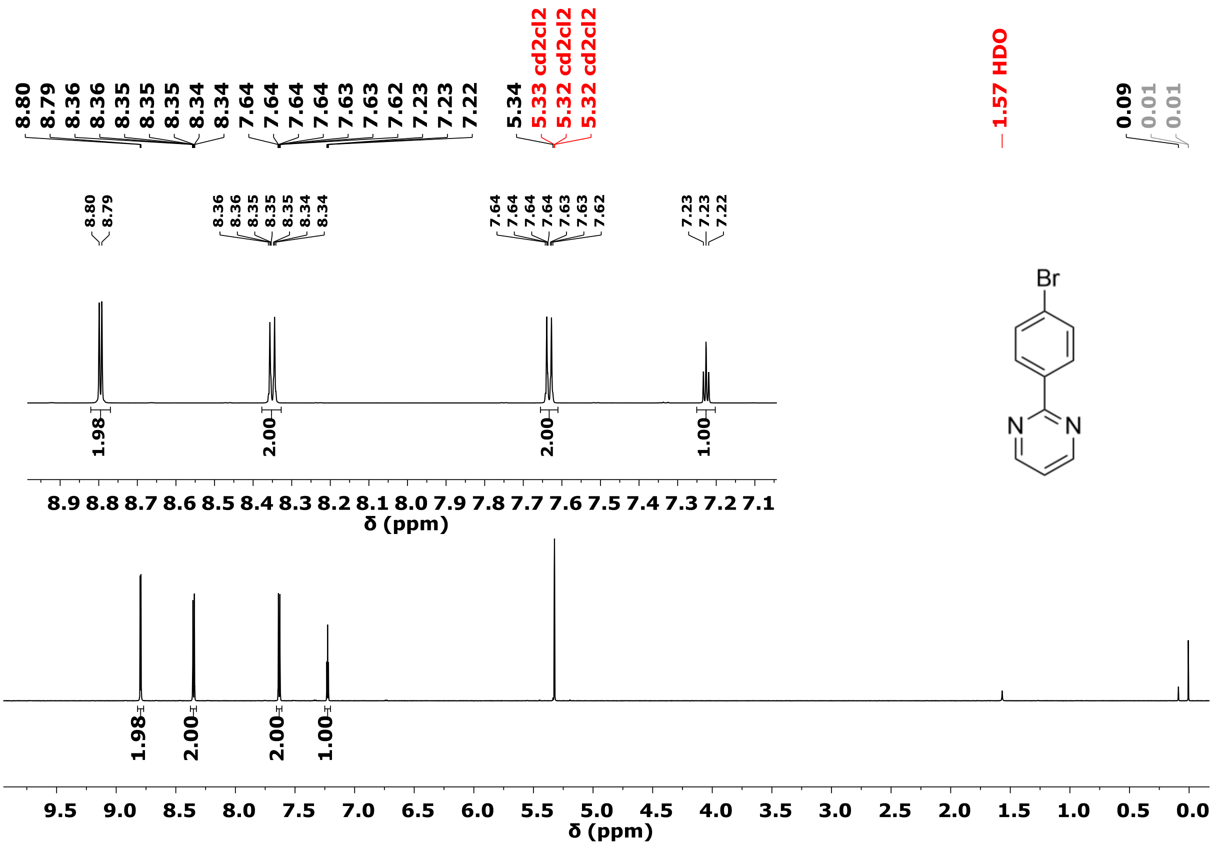

Figure S3: ^{1}H-NMR spectrum of **13** (CD_2Cl_2, 700 MHz, 298 K).

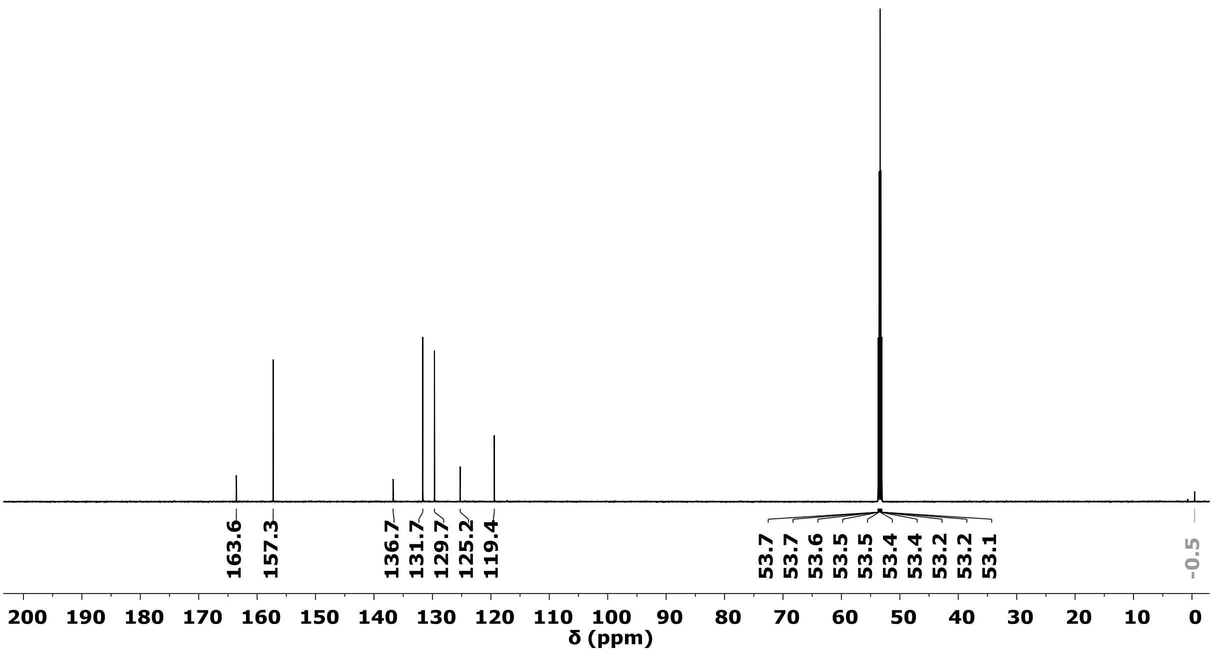

Figure S4: ^{13}C-NMR spectrum of **13** (CD_2Cl_2, 175 MHz, 298 K).

4.4. 2-(6-Bromopyridin-3-yl)pyrimidine 15

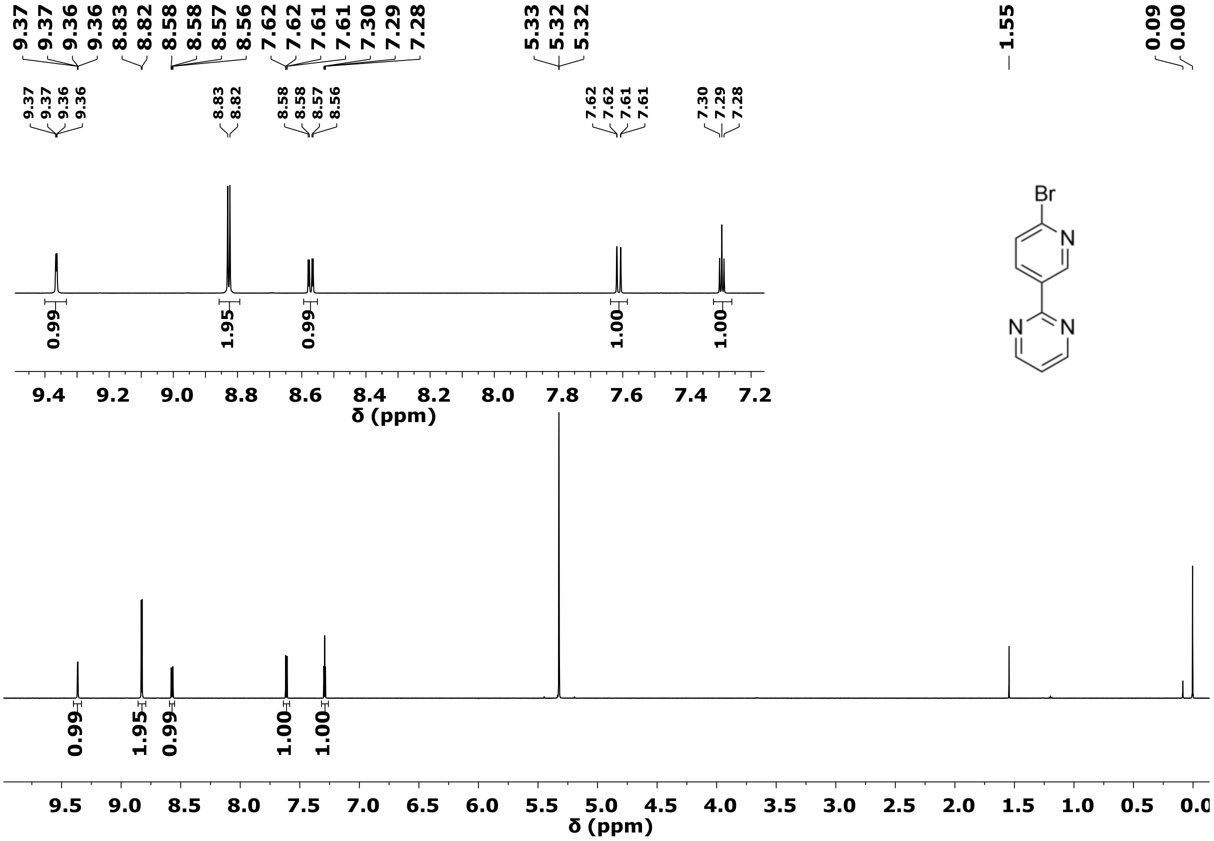

Figure S5: ^{1}H-NMR spectrum of **15** (CD_2Cl_2, 700 MHz, 298 K).

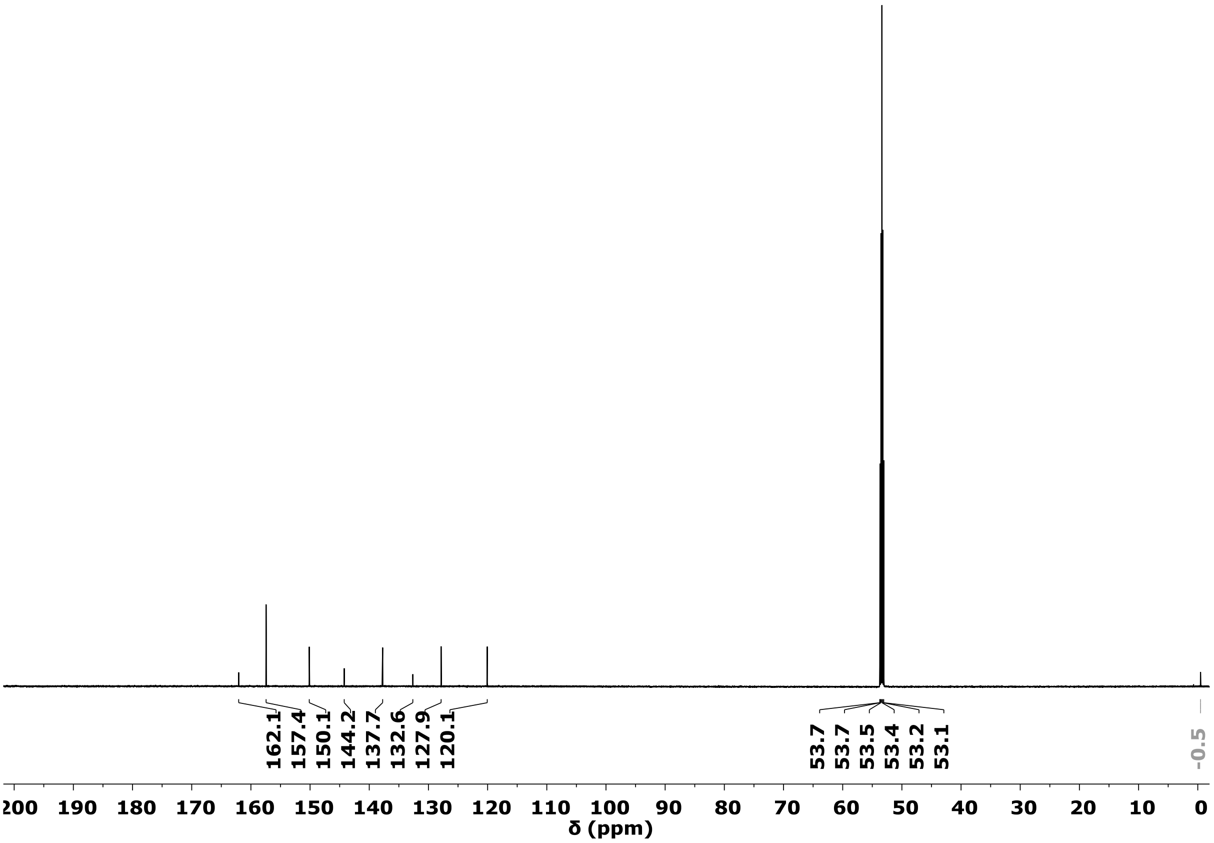

Figure S6: ^{13}C-NMR spectrum of **15** (CD_2Cl_2, 175 MHz, 298 K).

4.5. 6’-Bromo-2,3’-bipyridine 14

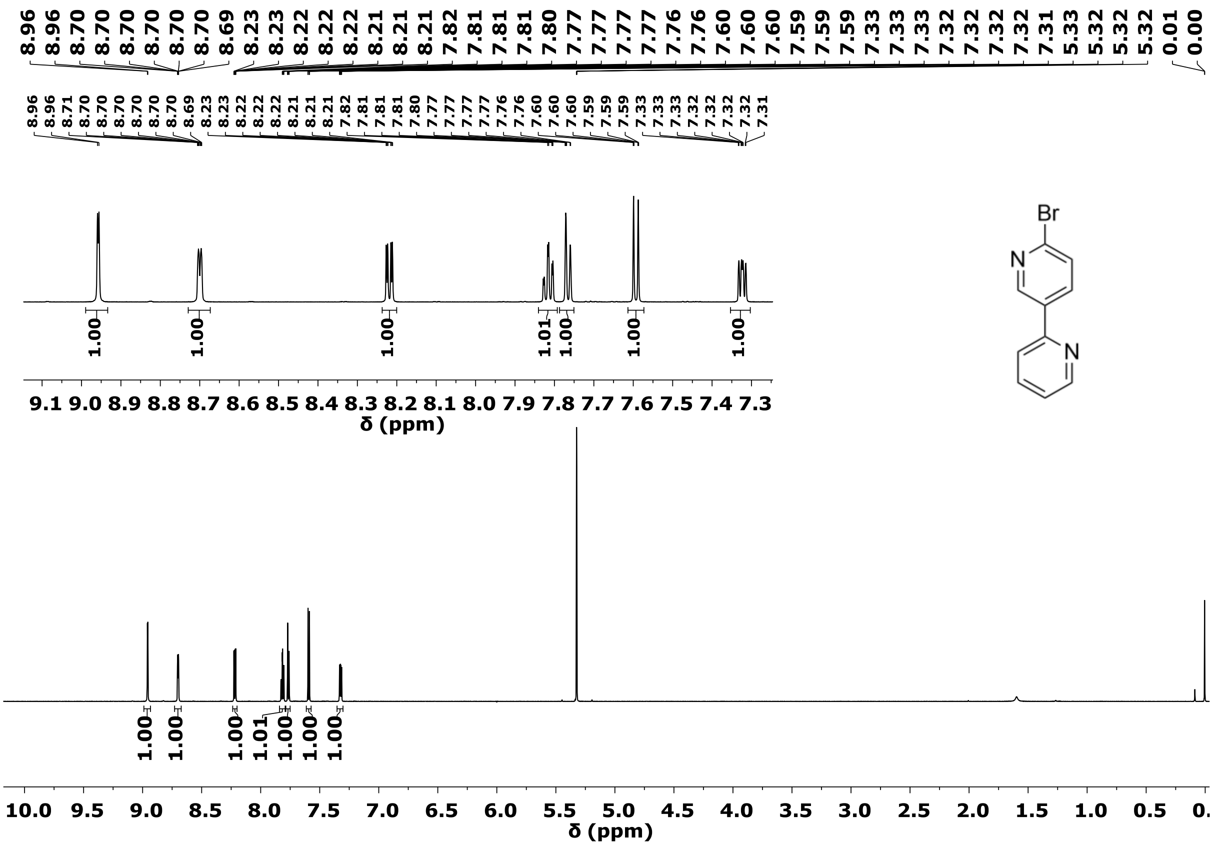

Figure S7: ^{1}H-NMR spectrum of **14** (CD_2Cl_2, 700 MHz, 298 K).

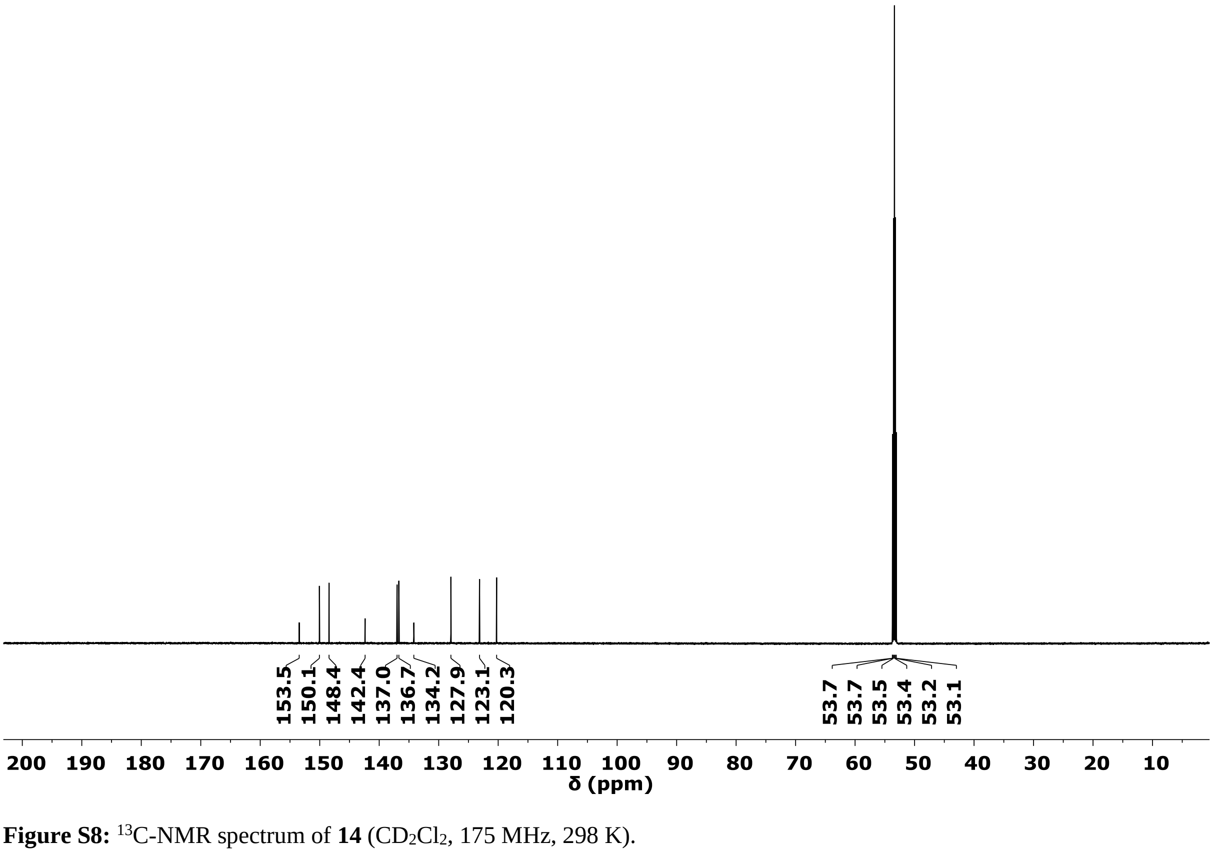

Figure S8: ^{13}C-NMR spectrum of **14** (CD_2Cl_2, 175 MHz, 298 K).

4.6. Compound 1

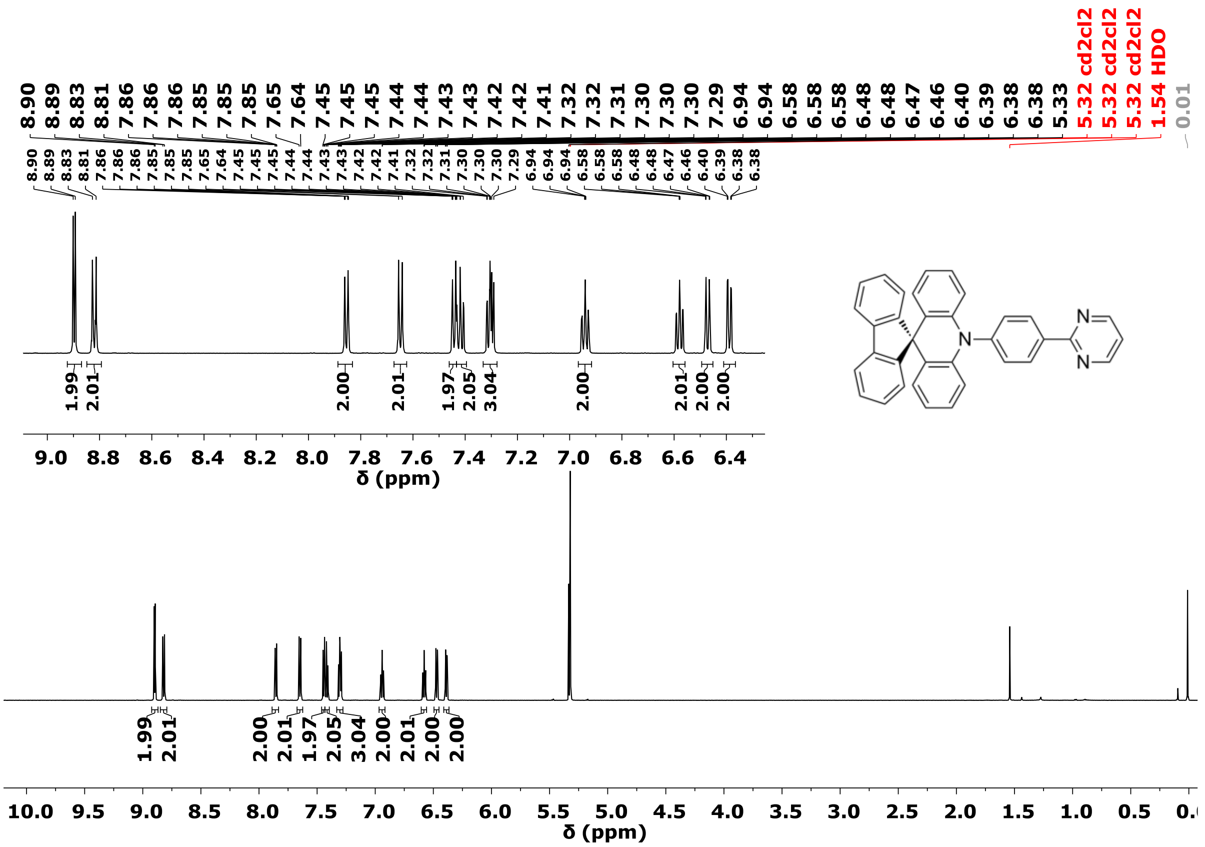

Figure S9: ^{1}H-NMR spectrum of **1** (CD_2Cl_2, 600 MHz, 298 K).

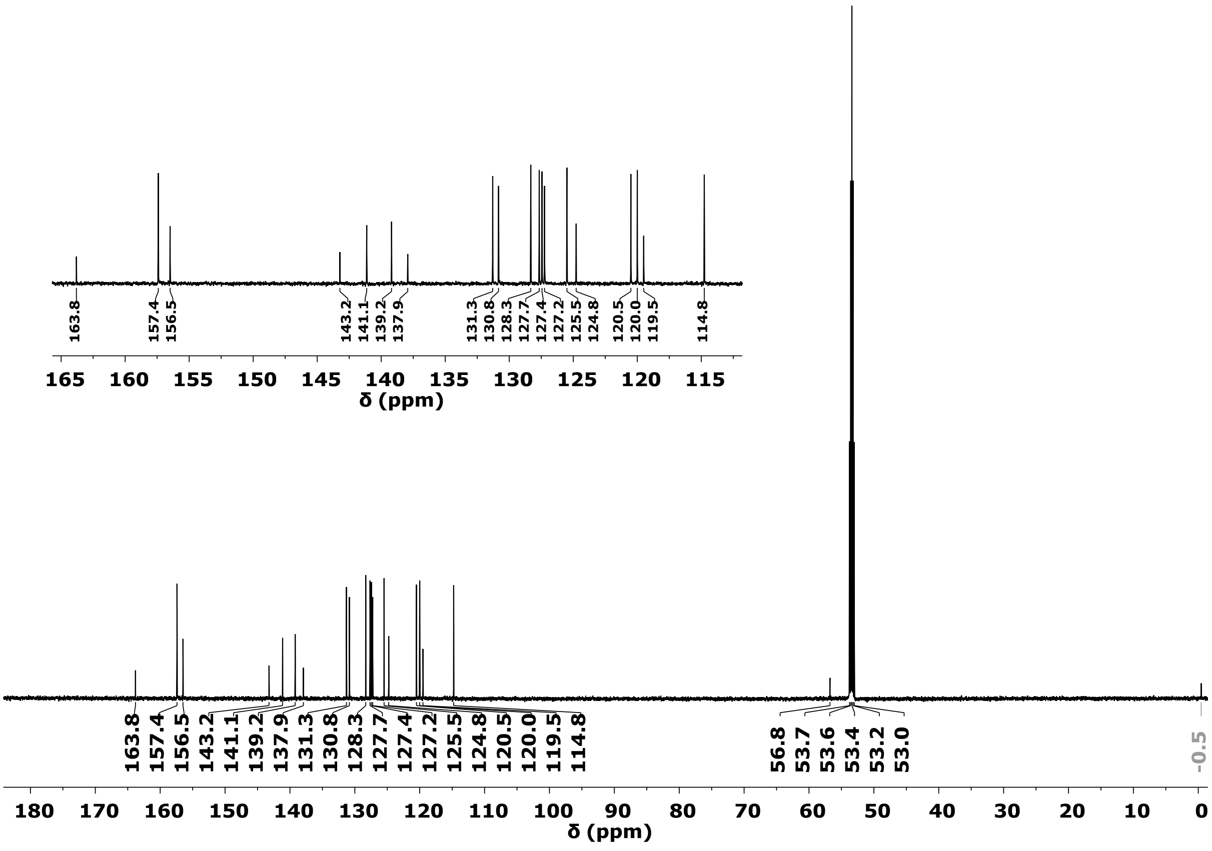

Figure S10: ^{13}C-NMR spectrum of **1** (CD_2Cl_2, 150 MHz, 298 K).

4.7. Compound 2

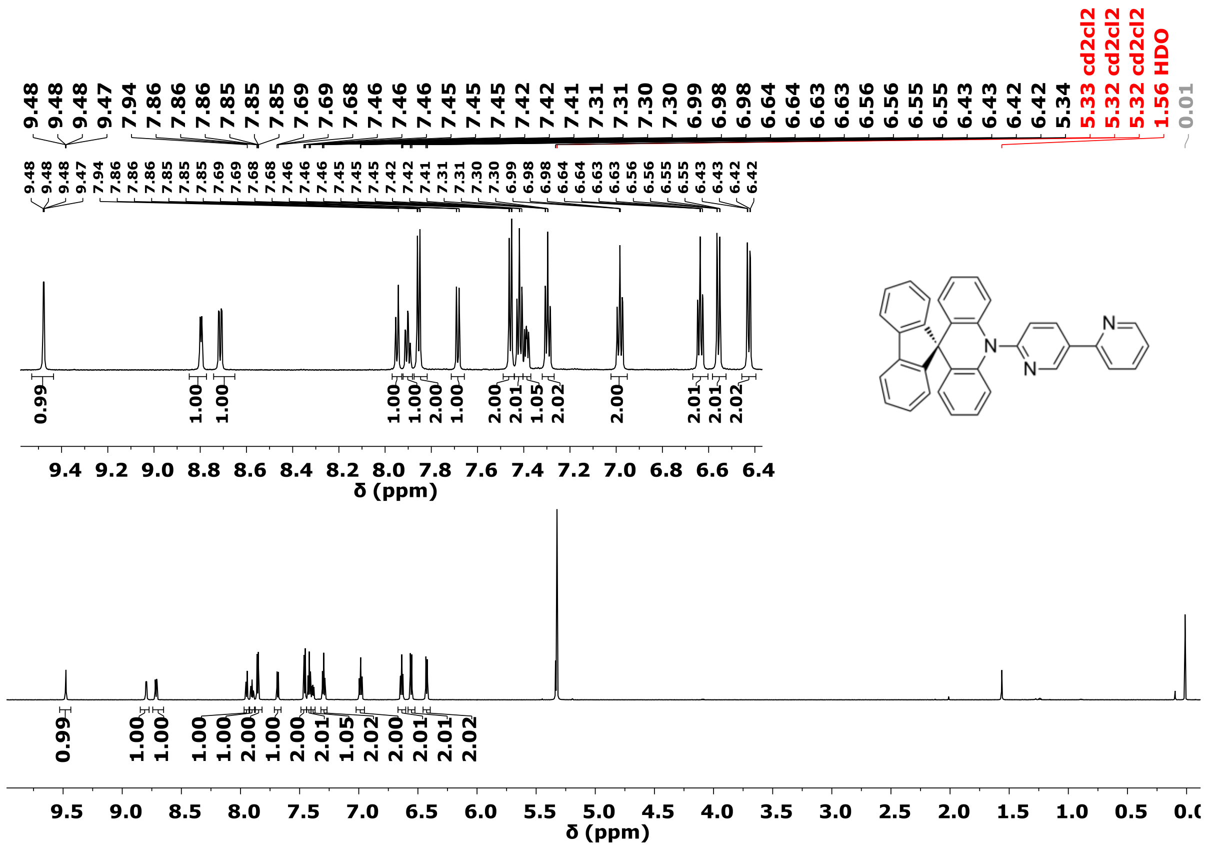

Figure S11: ^{1}H-NMR spectrum of **2** (CD_2Cl_2, 700 MHz, 298 K).

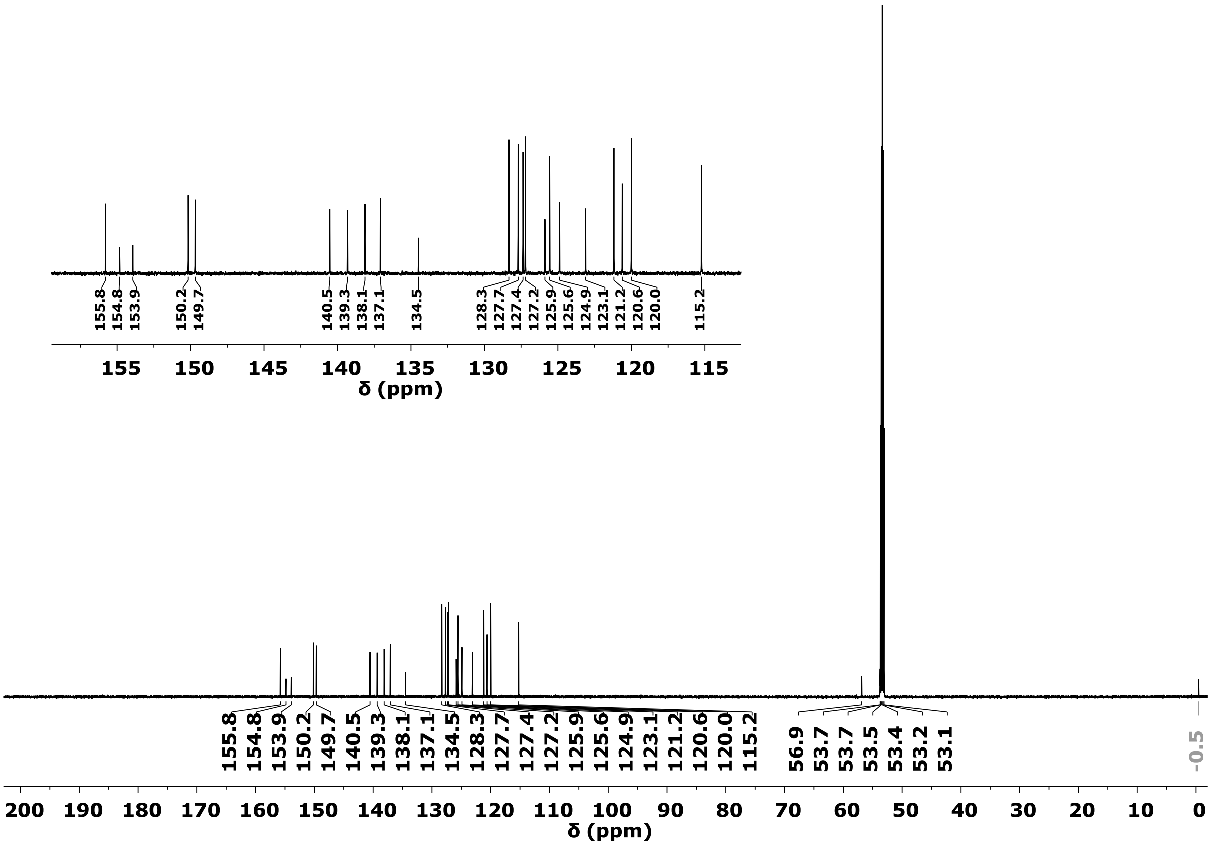

Figure S12: ^{13}C-NMR spectrum of **2** (CD_2Cl_2, 175 MHz, 298 K).

4.8. Compound 3

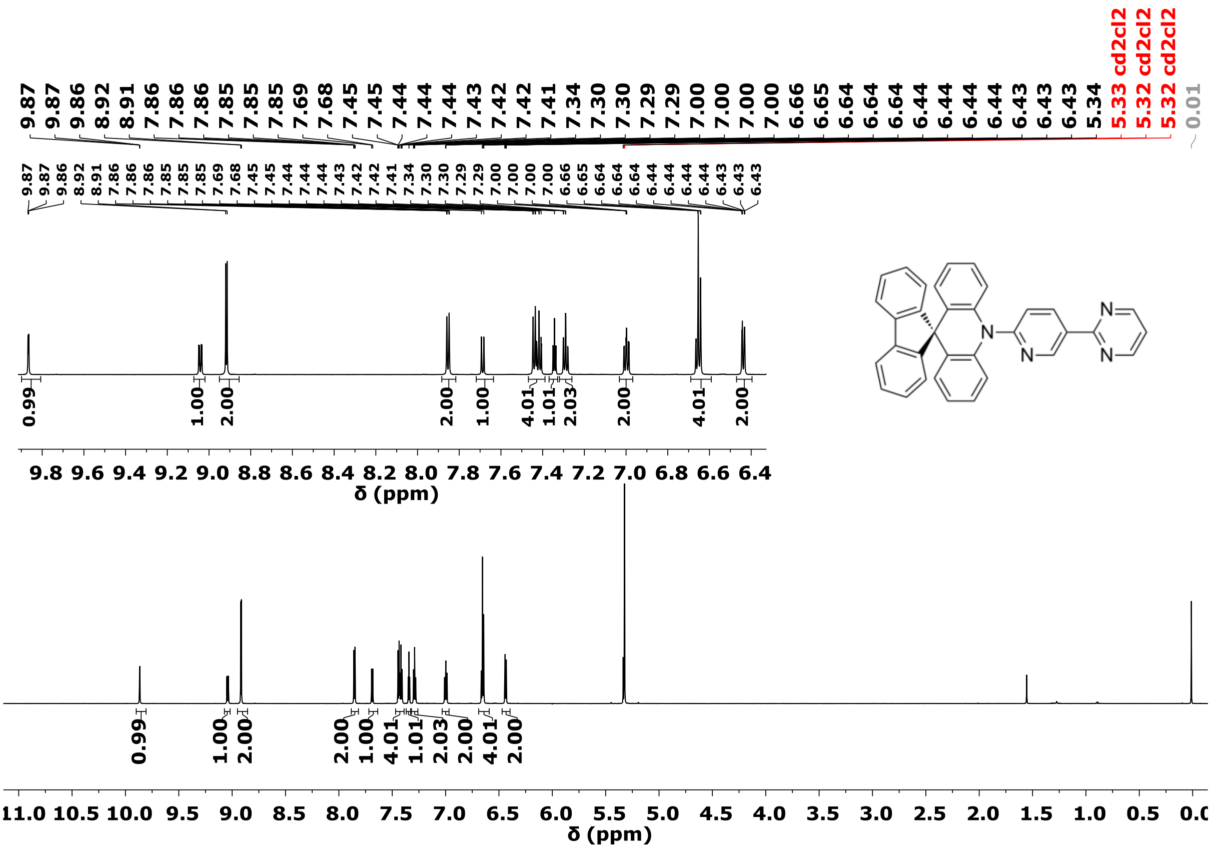

Figure S13: ^{1}H-NMR spectrum of **3** (CD_2Cl_2, 700 MHz, 298 K).

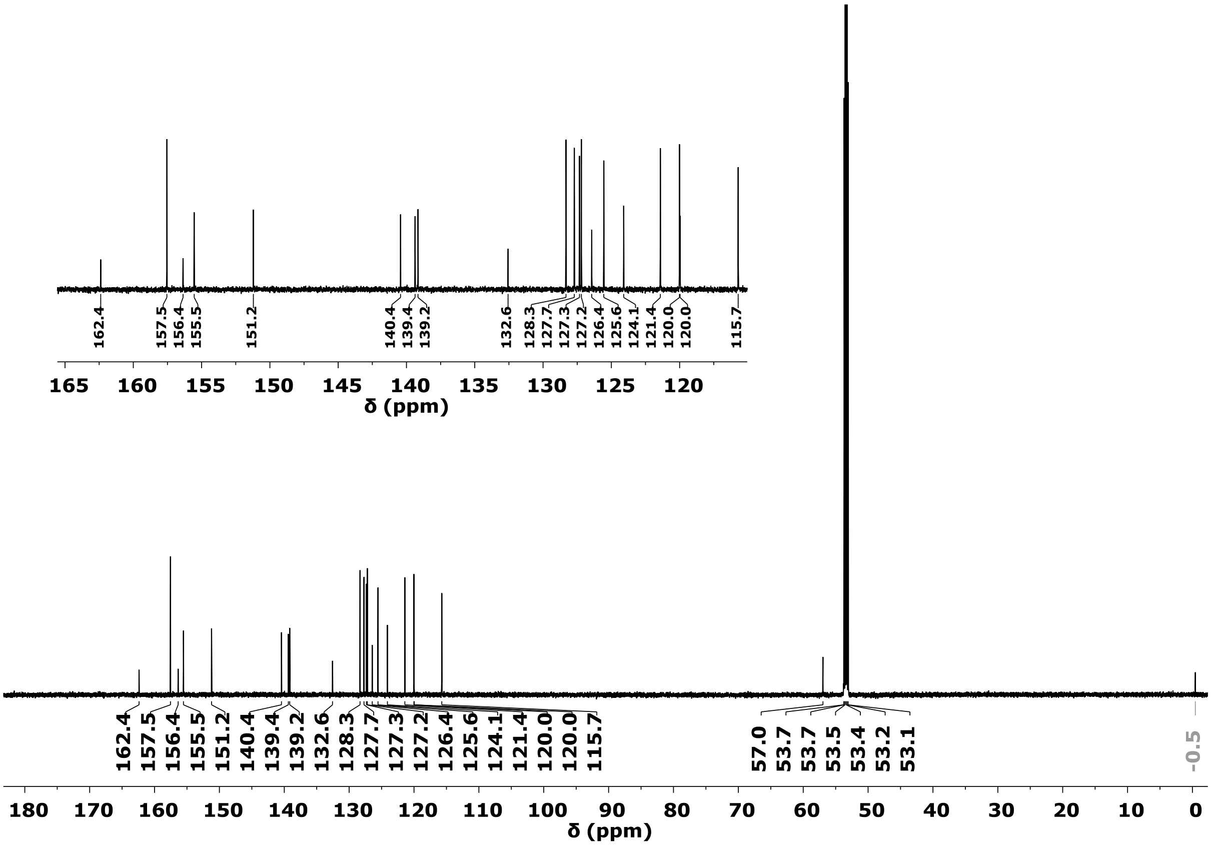

Figure S14. ^{13}C-NMR spectrum of **3** (CD_2Cl_2, 175 MHz, 298 K).

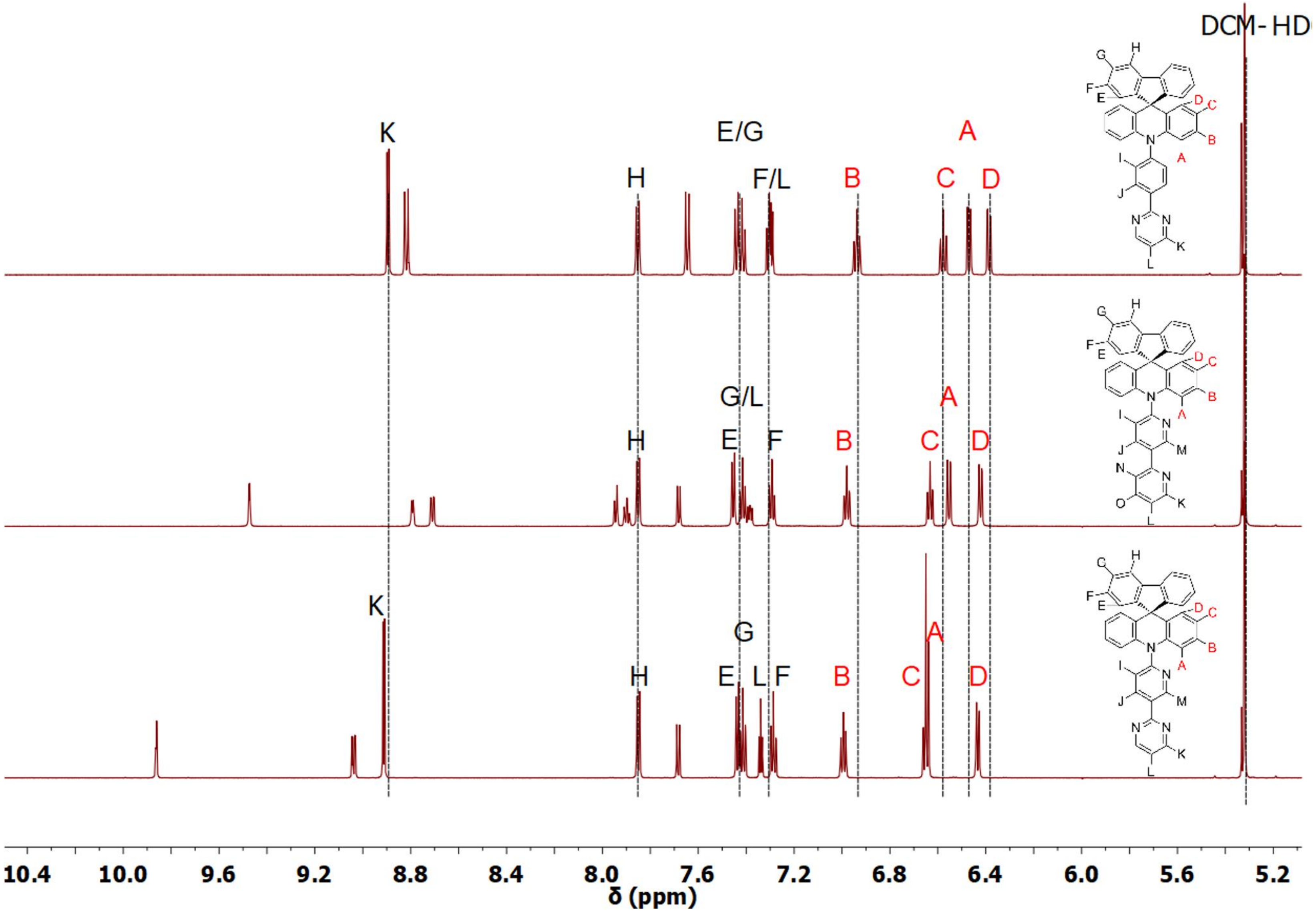

Figure S15. Comparison of ^{1}H-NMR spectra of **1** (top), **2** (center) and **3** (bottom) (DCM-d$_2$, 400 MHz, 298 K).

5. VT-NMR

To investigate on the structural dynamics of the three molecules, compounds **1-3** were probed by variable temperature ^{1}H-NMR experiments in DCM-d_2 solutions (Figures S16-S19). In the temperature range between room temperature and -80°C, no coalescence of proton signals or desymmetrization of the overall molecular structures could be observed, indicating no pronounced structural dynamic differences, enhanced intersegmental interactions or distinguishable rotational energy barriers in between the derivatives investigated. However, at lower temperatures (i.e. -80°C), the signals of the acridine protons of each compound show a more pronounced shift towards lower ppm, indicating a generally higher degree of acridine planarization at this temperature due to lower available thermal energy. Since the upfield shift of the acridine proton signals at -80°C is more pronounced in case of the pyridyl-containing compound **2** and **3** as it is for the unfunctionalized compound **1**, a greater structural flexibility of the acridine unit in **2** and **3** is evident. This effect could result from reduced steric interactions as a consequence of interchanging the bridging phenyl group of compound **1** for the pyridyl groups in materials **2** and **3**, but is not indicative for the occurrence of intramolecular H-bonding.

5.1. Compound 1

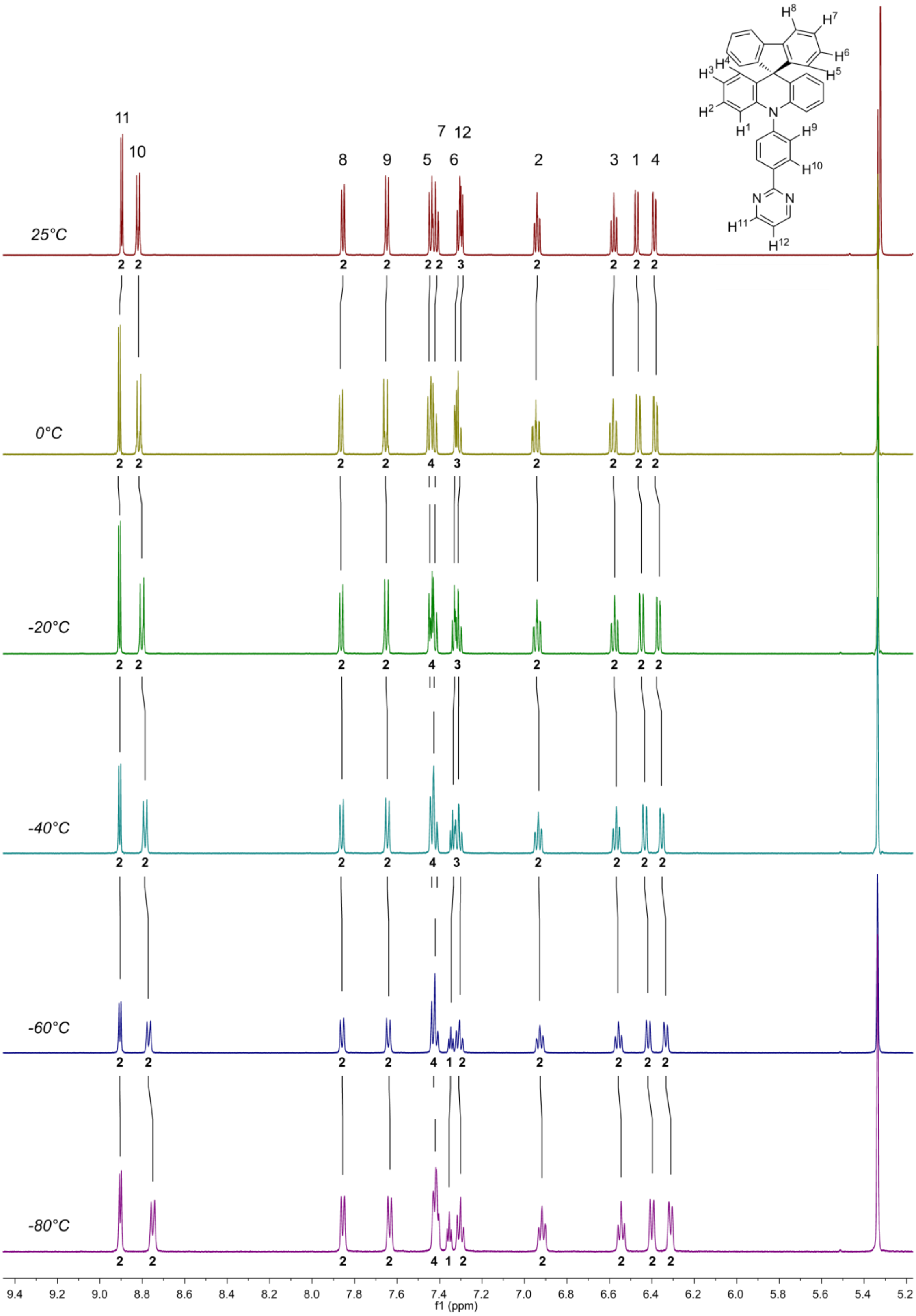

Figure S16. ^{1}H-NMR spectra of compound **1** at various temperatures (CD_2Cl_2, 400 MHz). Baseline numbers indicating the integral of the respective peak.

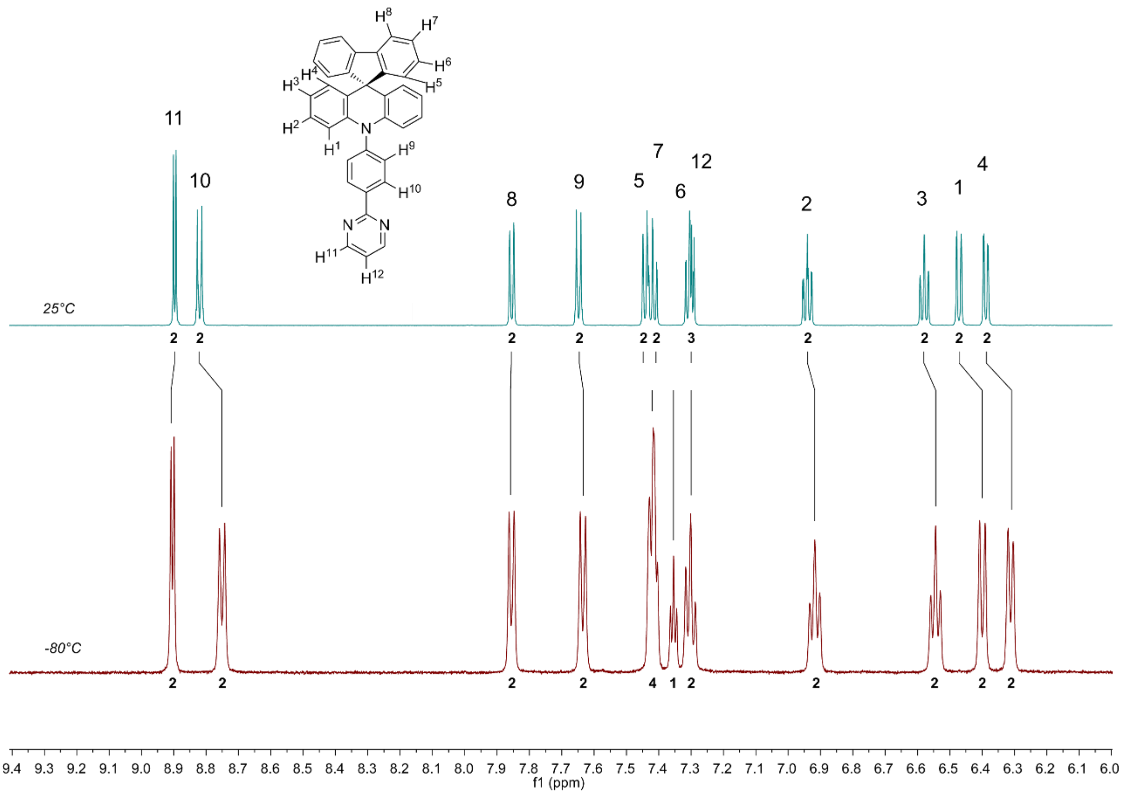

Figure S17. ^{1}H-NMR spectra of compound **1** at 25°C (top) and -80°C (bottom) (CD_2Cl_2, 400 MHz). Baseline numbers indicating the integral of the respective peak.

5.2. Compound 2

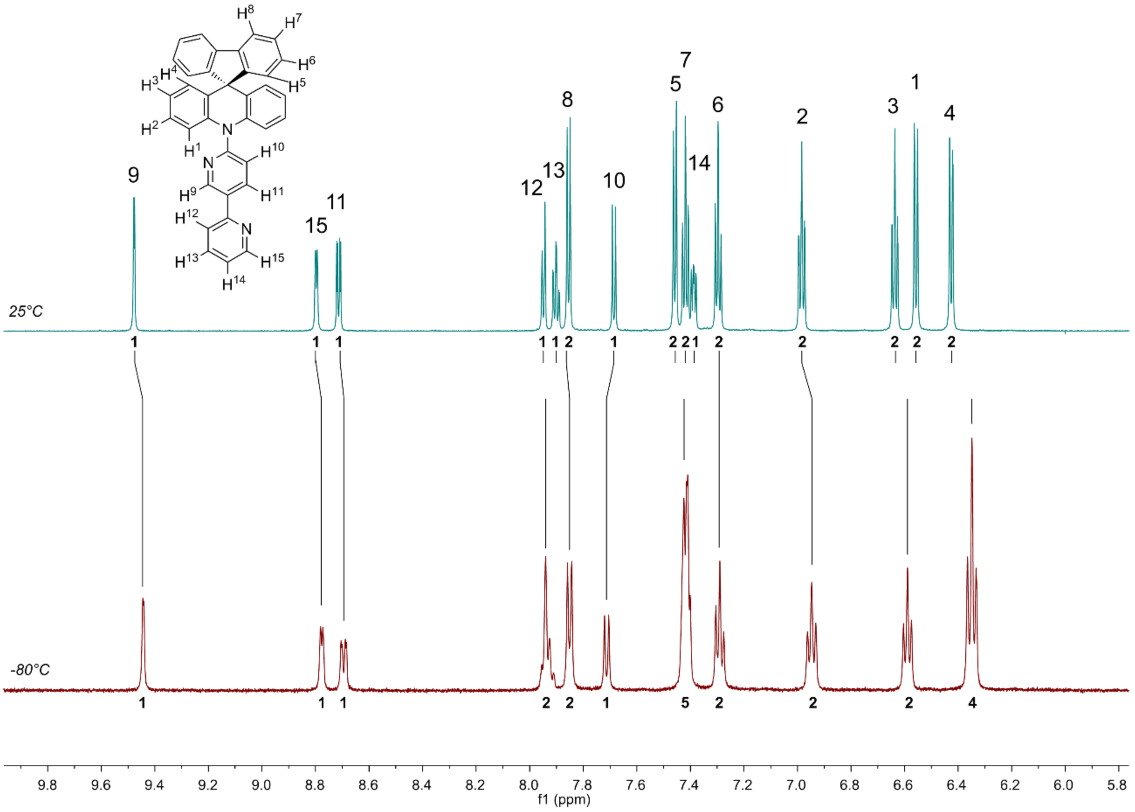

Figure S18. ^{1}H-NMR spectra of compound **2** at 25°C (top) and -80°C (bottom) (CD_2Cl_2, 400 MHz). Baseline numbers indicating the integral of the respective peak.

5.3. Compound 3

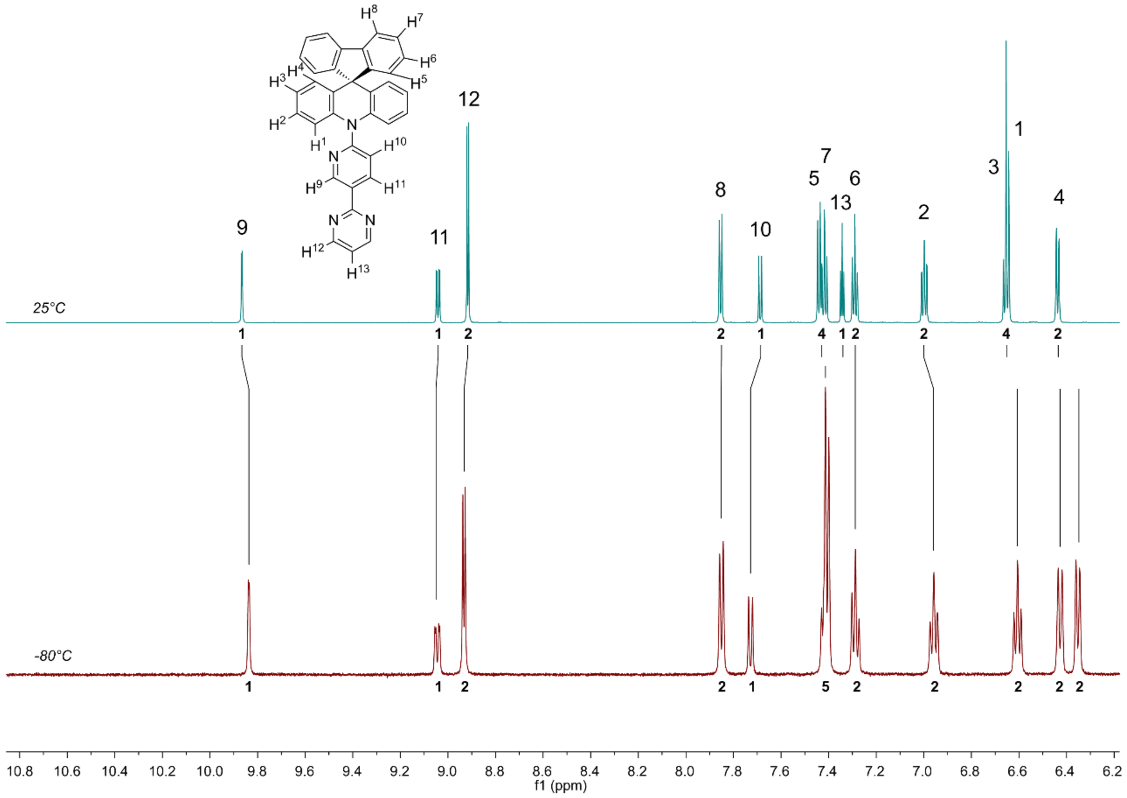

Figure S19. ^{1}H-NMR spectra of compound **3** at 25°C (top) and -80°C (bottom) (CD_2Cl_2, 400 MHz). Baseline numbers indicating the integral of the respective peak.

6. X-Ray Crystallography

Molecular structures of **1-3**, determined by single-crystal X-ray diffraction, are shown in Figure S20, crystal data and experimental details are listed in Table S2. It is noteworthy that although compounds **1** and **2** are isomers, the molecular volume of the latter is 3% smaller. Crystals of **2** and **3** are isostructural (isomorphous), in both the asymmetric unit comprising two molecules with practically identical conformations. In all three structures, molecules are arranged in centrosymmetric dimers with π-π stacking between donor and acceptor moieties (Figure S21). In structure **2**, all pyridyl N atoms participate in intermolecular C−H···N contacts of 2.58-2.70 Å (as calculated for C−H bond lengths adjusted to 1.08 Å), see Figure S22. While these contacts are only slightly shorter than the sum of van der Waals radii (2.74 Å)[26], they can be compared with the *ab initio* calculations of $CH_4 \cdot NH_3$ dimer[27] which gave H···N distance of 2.82 Å and bond dissociation energy of 2.5 kJ/mol. In structure **3**, only atoms N(32) and N(38) of each independent molecule (i.e. sterically the same N atoms as in **2**!) form C−H···N contacts of 2.53-2.81 Å, while N(34) forms none (Figure S23); this may explain why substitution of a pyrimidyl for a pyridyl group does not affect the crystal packing substantially. In structure **1**, intermolecular C−H···N(pyrimidyl) contacts are much longer, 2.90 Å for N(38) and 3.11 Å for N(34). Thus, competition between intermolecular and intramolecular hydrogen bonds, and the weakness of both, makes it difficult to ascribe the decisive influence to the intramolecular hydrogen bonding, at least from crystallographic data alone.

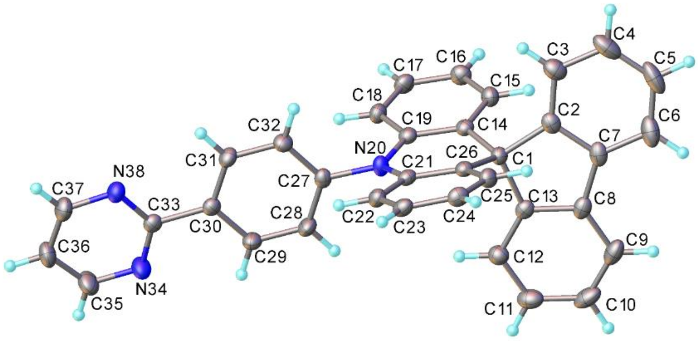

(**1**)

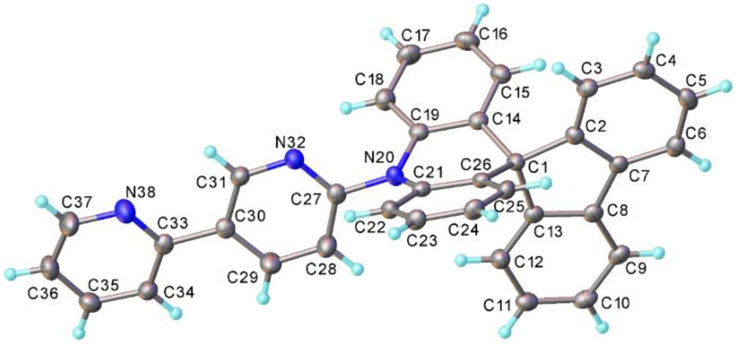

(**2**)

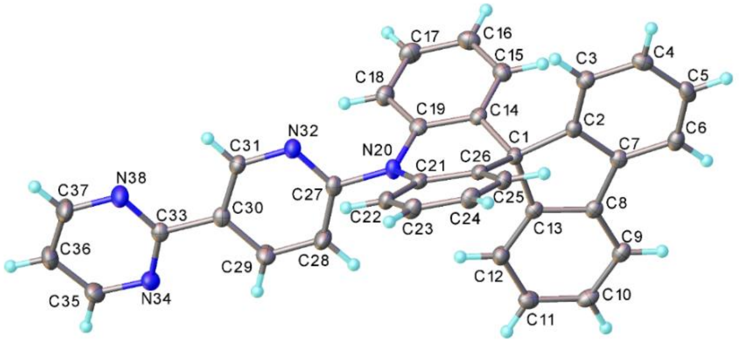

(**3**)

Figure S20. X-ray molecular structures of compounds **1** (crystallized from *n*-hexane/DCM solution), **2** and **3** (from DCM-d_2; one independent molecule shown) at 120 K. Atomic displacement ellipsoids are drawn at the 50% probability level.

Table S2. Crystal data and experimental details*

**Compound**	**1**	**2**	**3**
CCDC dep. no.	2159816	2159817	2159818
Formula	$C_{35}H_{23}N_3$	$C_{35}H_{23}N_3$	$C_{34}H_{22}N_4$
Formula weight	485.56	485.56	486.55
T/K	120	120	120
Crystal System	monoclinic	triclinic	triclinic
Space Group	$P2_1/c$ (no. 14)	$P\bar{1}$ (no. 2)	$P\bar{1}$ (no. 2)
a/Å	9.1720(3)	9.3676(3)	9.3372(3)
b/Å	16.3241(6)	10.8168(4)	10.8424(4)
c/Å	16.8889(6)	24.6571(9)	24.4587(9)
α/°	90	99.0423(14)	99.2192(14)
β/°	103.4439(15)	95.7251(14)	95.8775(16)
γ/°	90	102.9213(14)	102.8324(14)
V/Å^3	2459.4(2)	2381.2(2)	2358.5(2)
Z	4	4	4
$D_{calc.}$/ g cm^{-3}	1.311	1.354	1.370
μ/mm^{-1}	0.08	0.08	0.08
$2\theta_{max}$/°	50	50	55
Reflections total	37256	36562	44596
unique	4324	8356	10806
with $I > 2\sigma(I)$	3404	6183	8392
R_{int}	0.057	0.044	0.061
Refined parameters	344	686	686
$\Delta\rho$ /eÅ^{-3}	0.20, -0.16	0.28, -0.18	0.36, -0.23
Goodness of fit	1.025	1.016	1.021
R_1, wR_2 (all data)	0.079, 0.086	0.090, 0.100	0.104, 0.113
R_1, wR_2 [$I > 2\sigma(I)$]	0.035, 0.053	0.040, 0.065	0.044, 0.062

*Mo-$K\alpha$ radiation, λ = 0.71073 Å

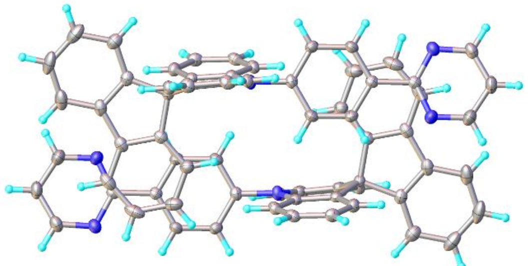

Fluorene-acceptor π−π overlap

	interplanar angle, deg	mean separation, Å
**1**	12.0	3.82
**2 a-a**	8.2	3.54
**2 b-b**	10.5	3.56
**3 a-a**	10.1	3.54
**3 b-b**	12.6	3.54

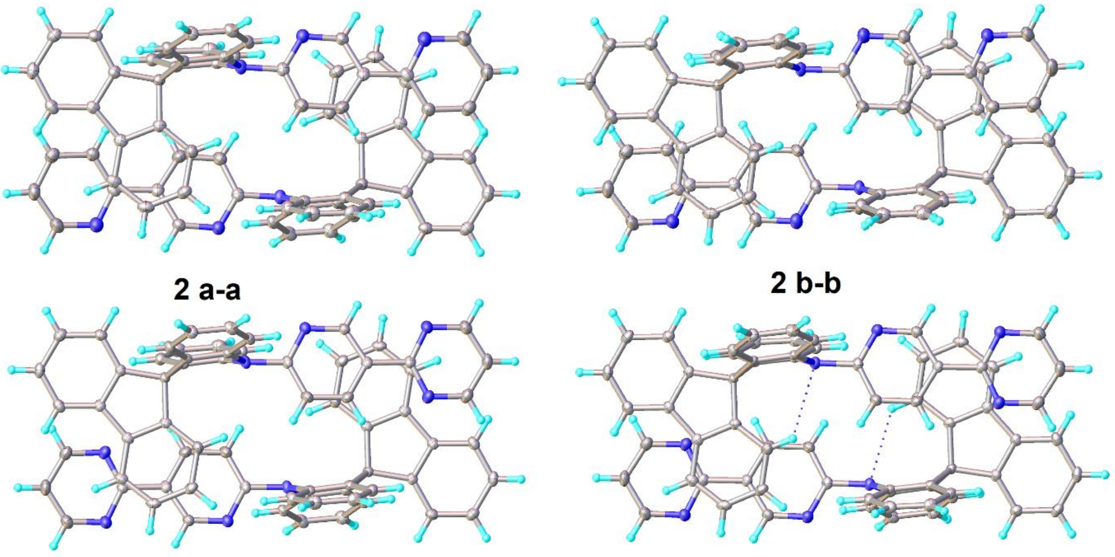

Figure S21. Dimers in the structures of **1-3**

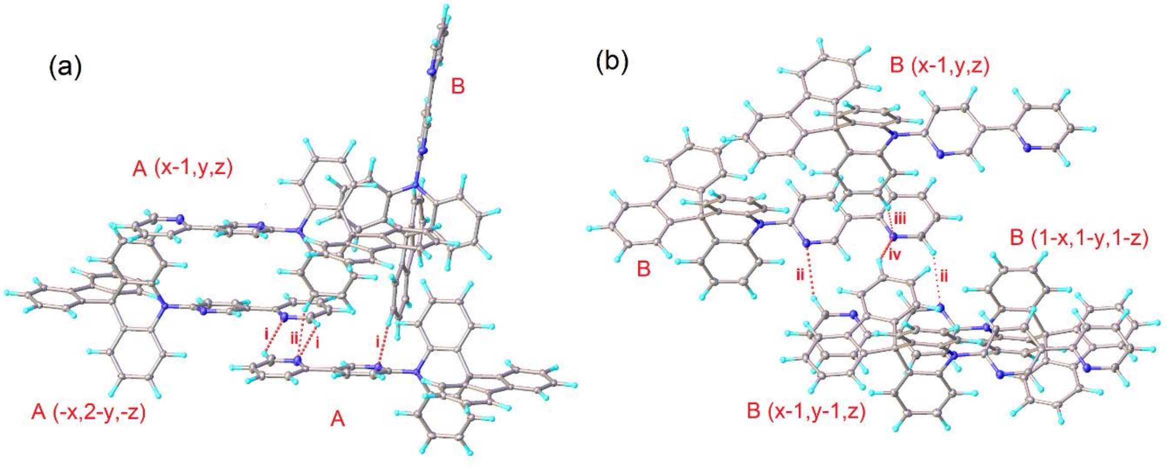

Figure S22. Intermolecular C−H⋯N contacts around molecules A (a) and B (b) in the crystal of **2.** H⋯N distances: (i) 2.58, (ii) 2.68, (iii) 2.60, (iv) 2.70 Å.

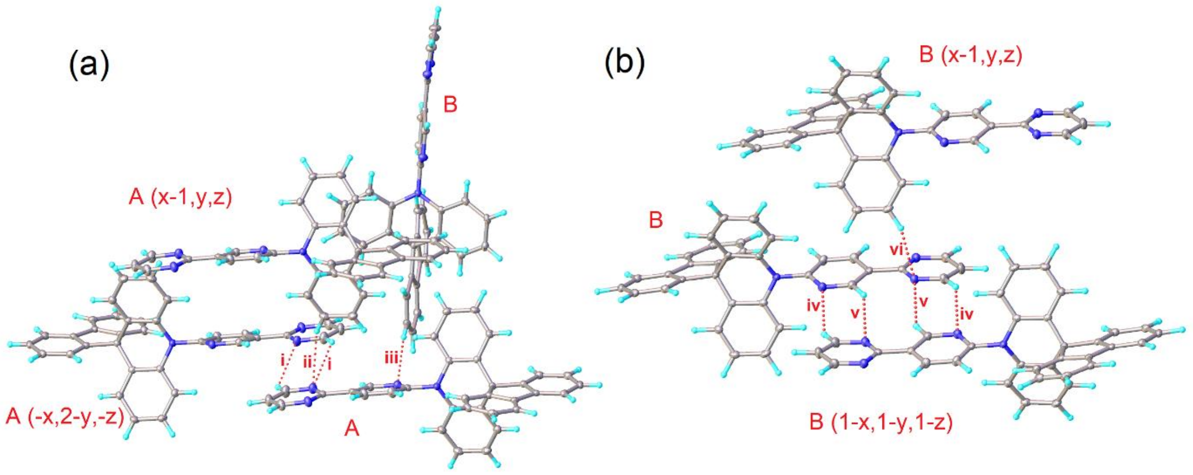

Figure S23. Intermolecular C−H⋯N contacts around molecules A (a) and B (b) in the crystal of **3.** H⋯N distances: (i) 2.76, (ii) 2.79, (iii) 2.53, (iv) 2.58, (v) 2.59, (vi) 2.71 Å.

7. Photophysical properties

In order to get an insight into the optical and photophysical properties of the spiroacridine derivatives, steady-state UV-Vis absorption and photoluminescence (PL) in the non-polar, optically transparent Zeonex matrix were recorded (Figure 2). Because of the low concentration and relatively fluid nature of the polymer host, we do not expect any aggregation or packing effects in these films. While both **1** and **2**, containing two nitrogen atoms in their electron-accepting segments, absorb until 420 nm, the UV-Vis spectrum of **3** extends until 440 nm. More red-shifted low-energy onset in the absorption of **3** is in line with the lower LUMO energy level and higher CT strength in this compound. Interestingly, the lower energy bands (LEB) of **2** and **3** consists of two overlapping bands peaking at 350 / 375 nm for **2** and 356 / 380 nm for **3**, respectively. While the lower intensity, broader parts of the LEBs correspond to the $S_0 \rightarrow S_1$ excitation of the CT nature (in agreement with the HOMO/LUMO analysis, **Section 8**), the blue-shifted higher intensity part is likely to be $S_0 \rightarrow S_2$ transition with the higher share of the electron density of the particle (Figure S30) localized on the pyridine spacer. Overall, the CT band onset red-shifts in the following order: **1** < **2** < **3**.

Photoluminescence (PL), recorded for the Zeonex films, confirms the tendencies observed in the absorption spectra. While the emission spectra of **1** and **2** almost overlap, the PL of the former has a distinct vibronic fine structure with 0→0 peak at 410 nm and 1→0 peak at 423 nm (Figure 2). The emission of **2**, on the other hand, is of a close to Gaussian shape with the slightly more red-shifted 0→0 peak at 414 nm and a 1→0 shoulder at 429 nm. Introduction of the additional nitrogen into the acceptor of **3** results, as expected, in a much more red-shifted PL peaking at 437 nm. However, we must note, that the difference in the CT character of **1** and **2** is so small, that the shifting order reverses in the media of different polarity (Figure S24).

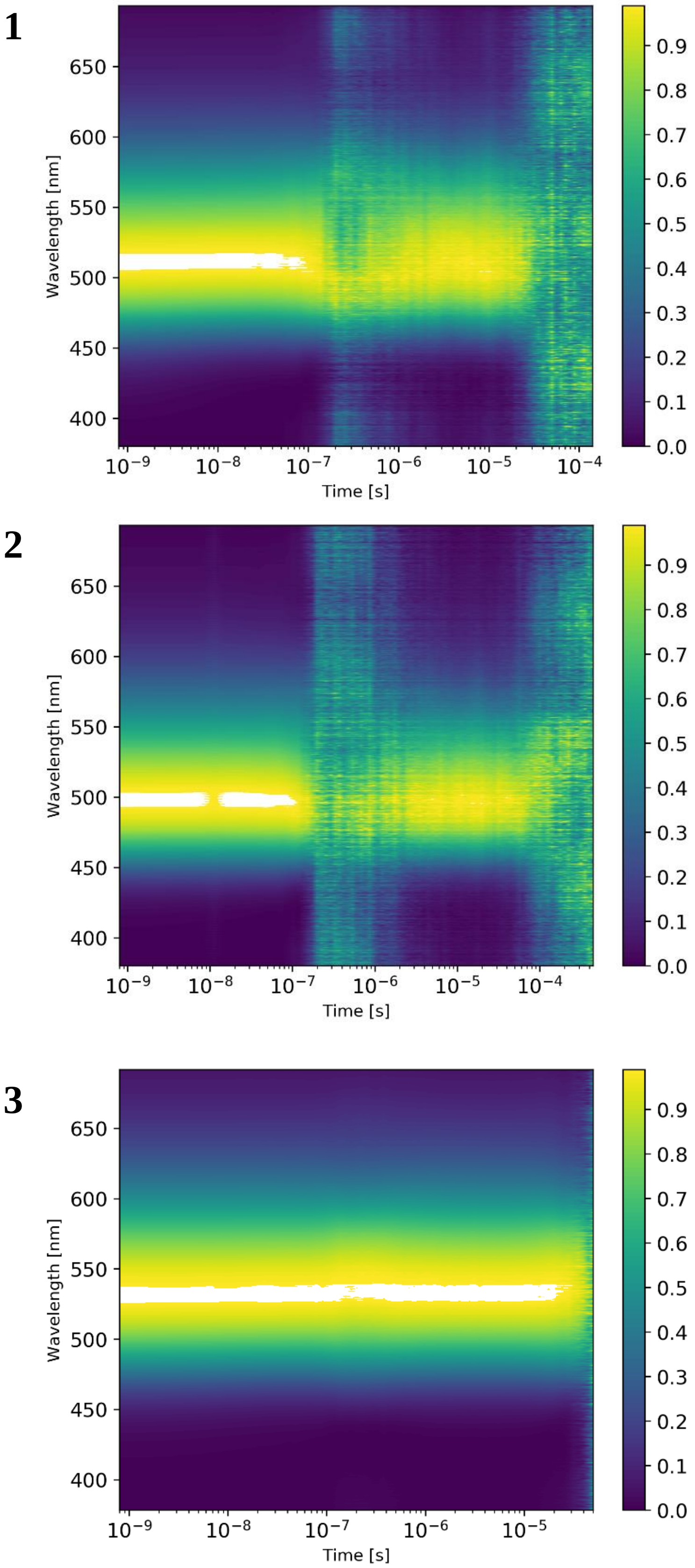

Figure S24. Contour plots of normalised time-resolved emission spectra of **1** (top), **2** (middle), and **3** (bottom) in degassed DCM solution (1 mgmL^{-1}). The absence of spectral shifts between PF and DF emission regimes (in contrast to Zeonex films) indicates an absence of RTP emission.

Similar shape of the phosphorescence spectra (Figure 2) and the coinciding onsets at 2.84-2.86 eV are in line with the similar T_1 nature, predicted by the NTO analysis (Figure S28).

Relatively large singlet/triplet energy gap in Zeonex films of **1** and **2** (0.34 eV) resulted in mixed delayed fluorescence and RTP evolving from blue to green over time (time delay 1-40 ms) in **1** and **2** (Figure S25 (a, b, d)). Interestingly, the presence of the pyridyl spacer in **2** leads to the longer prompt fluorescence (PF) lifetime of 7.6 ns as compared to 4.7 ns in **1**. The presence of three nitrogen atoms in the acceptor unit of **3** leads to a much more pronounced CT character even in Zeonex matrix and ΔE_{ST} of 0.16 eV. A smaller ΔE_{ST} translates in turn into the easily identifiable DF peaking at 462 nm (Figure S25 (c, d)). Similarly to the other materials, the RTP of **3** evolves from blue to green over time. As expected, only PF and phosphorescence were observed at 80K (Figure S27).

The presence of DF in **3**, containing the pyridinyl spacer, clearly points at the high CT character in this material, achieved by the stabilization of the near-orthogonal D-A geometry in the excited state. Ultimately none of these results can conclusively determine the presence or absence of intramolecular H-bonding in these materials though, as any differences in properties can also be entirely explained from differences in acceptor strength.

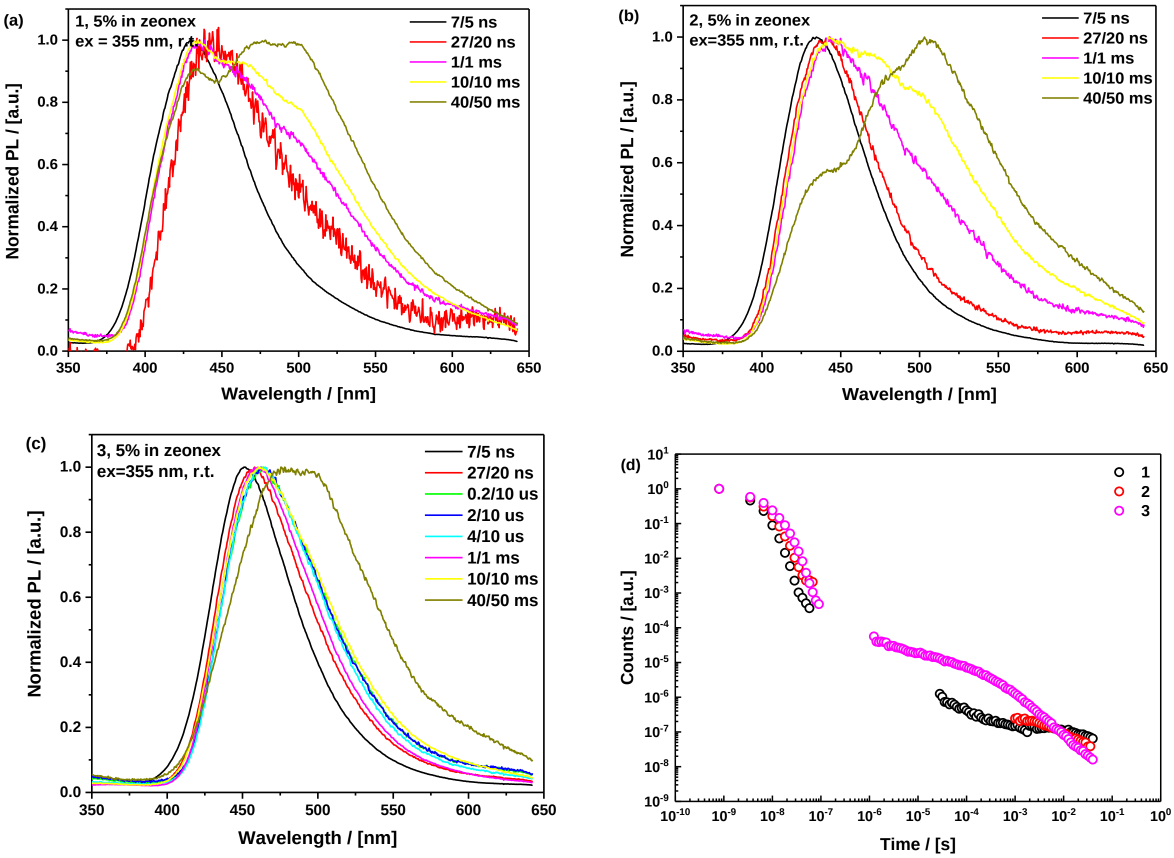

Figure S25. Time-resolved photoluminescence recorded for the 5% Zeonex films of (a) **1**, (b) **2** and (c) **3** at room temperature (ex = 355 nm) at specific delay and integration times (delay/integration); (d) decay curves of the 5 wt% Zeonex films of **1**, **2** and **3**.

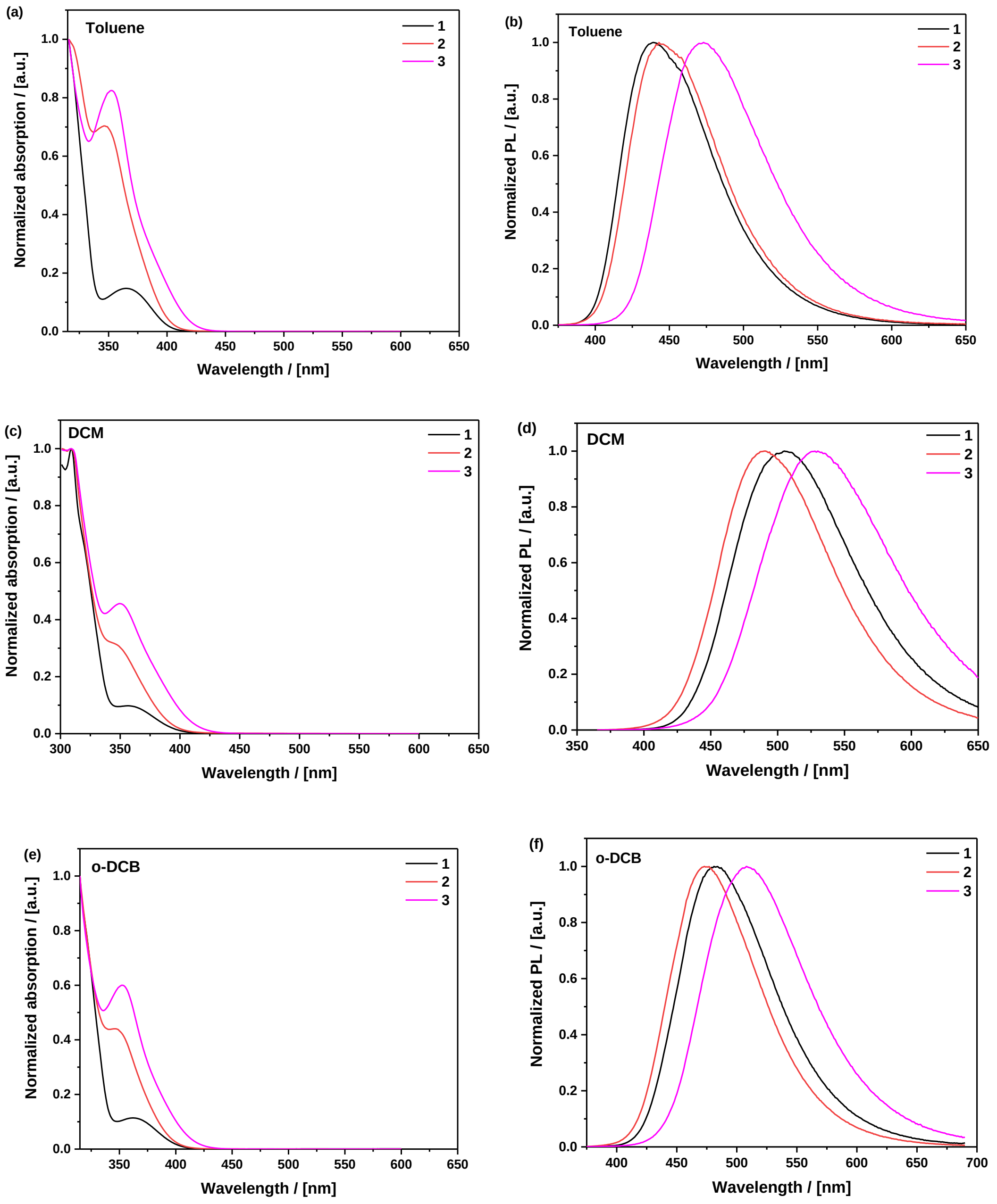

Figure S26. Steady-state (a, c, e) absorption and (b, d, f) photoluminescence (ex = 330 nm) of the 1 mgmL^{-1} solutions of **1**, **2** and **3** in various solvents.

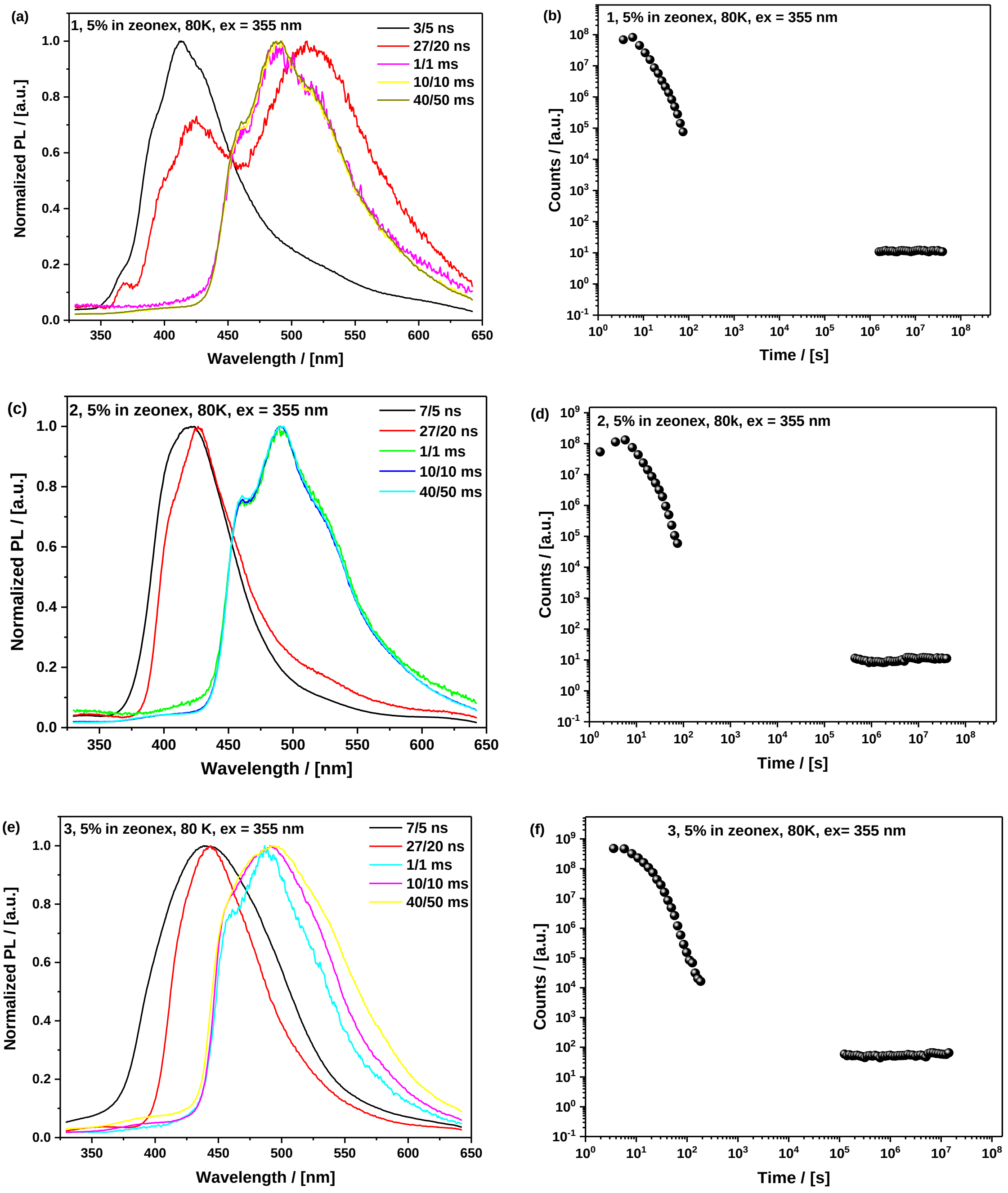

Figure S27. (a, c, e) Time-resolved photoluminescence recorded for the 5% Zeonex films of **1**, **2** and **3** at 80K (ex = 355 nm) at specific delay and integration times (delay/integration); (b, d, f) decay curves of the 5 wt% Zeonex films of **1**, **2** and **3**.

8. Theoretical calculations

The optimized ground state geometry of **1-3** was accessed at the rCAM-B3LYP/6-31G(d) level of theory (19% HF) (Figure 3).[28, 29] Utilization of the acridine donor, featuring a six-membered ring, results in a dihedral angle between the D and A chromophores in the S_0 state that is close to orthogonal (85-87°) in the three molecules as previously reported using BMK and CAMB3LYP functionals.[30, 31] Interestingly, introduction of the nitrogen atom to the *ortho*-position adjacent to the donor phenyl (compounds **2** and **3**) leads to significant structural changes, when compared to compound **1**. Thus, not only is the acridine fragment pre-folded by 19° in both **2** and **3**, as opposed to a planar structure in **1**, but also the acceptor moiety is tilted by 29° relatively to the plane of the acridine donor (Figure 3). Such cumulative folding and twisting can occur presumably due to the shift of the moment of inertia of the molecule upon breaking the symmetry by the introduction of the heteroatom. An asymmetrical structure is additionally supported by the weak electrostatic interaction between the nitrogen of the pyridine unit and the *ortho*-hydrogen of spiro-fluorene (2.85 Å). Note, that no such interaction was observed in **1** (the distance between the *ortho*-H of spirofluorene and the *ortho*-H of the phenyl ring is 4.23 Å).

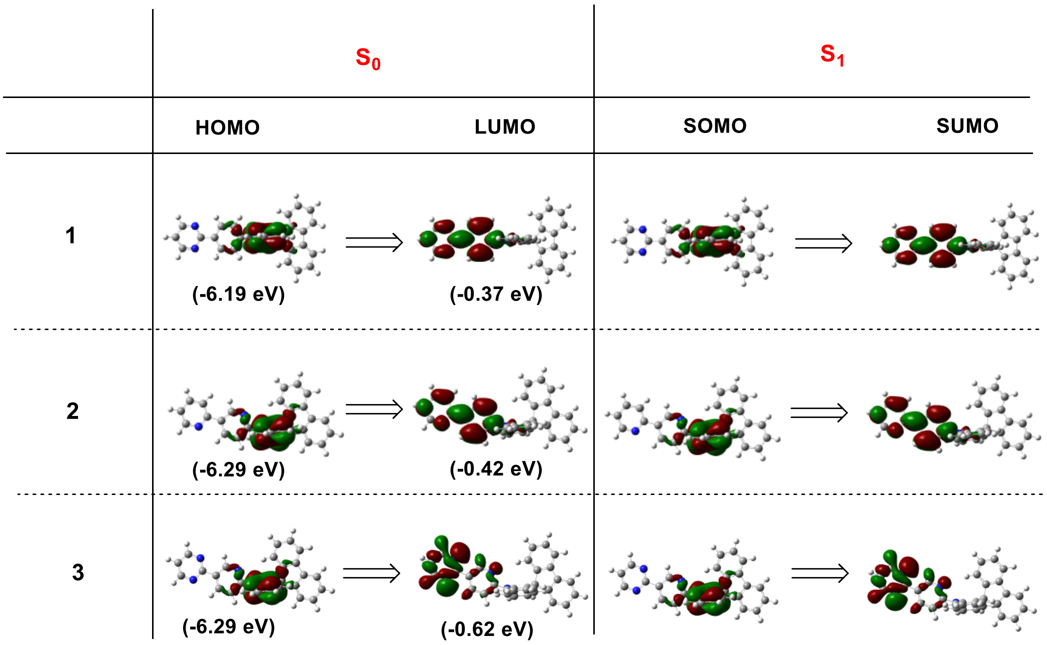

Figure S28. Frontier orbitals of ground state (rCAM-B3LYP/6-31G(d)) along with HOMO/LUMO energy values, and excited state (TDA-DFT CAM-B3LYP /6-31G(d)) of **1**, **2** and **3**. SOMO = Singly Occupied Molecular Orbital; SUMO = Singly Unoccupied Molecular Orbital.

Perpendicular geometry, in turn, translates into the well decoupled frontier orbitals (Figure S28), with a negligible HOMO/LUMO overlap observed in the case of the three compounds. It is noteworthy that both the acridine folding and the acceptor twisting affect the degree of HOMO/LUMO overlap. While the acridine-localized HOMO of **1** extends slightly onto the adjacent phenyl ring of the acceptor unit, the HOMOs of **2** and **3** delocalize additionally over the central part of the spirofluorene moiety. The LUMO of compound **1** is solely localized on the electron acceptor, the LUMO of **2**, featuring two redistributed nitrogens on the accepting fragment, extends onto the acridine (Figure S28). Three

electron-accepting nitrogens in **3** in conjunction with pre-twisted acceptor and pre-folded acridine donor, lead to the mainly pyrimidine-localized LUMO, with additional electron density on the neighboring pyridine's nitrogen. Evidently, these differences translate into the various frontier orbital values: the planar skeleton of **1** results in the highest HOMO (-6.19 eV) and LUMO (-0.37 eV), with the HOMO lowering to -6.29 eV in **2** and **3** due to acridine folding assisting the interruption of conjugation. This conjugation disturbance results in a slightly lower LUMO of **2** (-0.42 eV) as compared to **1** (-0.37 eV), despite of the equal number of electron-accepting nitrogens. Predictably, the introduction of an additional nitrogen into the pre-twisted acceptor of **3** lowers the LUMO even further (-0.62 eV). Of note, the HOMO and LUMO values are underestimated due to the gas phase calculations, and are presented here with a sole goal of depicting the structural differences between the materials.[32]

The geometry of **1-3** remains practically unchanged in the S_1 state (Figure 3), which results in a similar trend in the singly occupied molecular orbital (SOMO) and the singly unoccupied molecular orbital (SUMO) (Figure S28), as was observed for the ground state.

Theoretical UV/Vis absorption spectra of the spiroacridine derivatives display two major peaks and extend to 322-346 nm (Figure S29). However, subtle structural differences translate in the peak intensity differences and the variation in the charge transfer (CT) strength in these materials. Thus, the higher intensity of the first peak in **1** can be assigned to the π-π* absorption of the acceptor's phenyl ring, which is swapped to pyridine in **2** and **3**. Furthermore, while the zero-oscillator strength $S_0 \rightarrow S_1$ transition of **1** peaks at 322 nm, the introduction of the ortho-nitrogen red-shifts the absorption and enhances the oscillator strength (λ = 332 nm, f = 0.0017 for **2**; λ = 346 nm, f = 0.0016 for **3**).

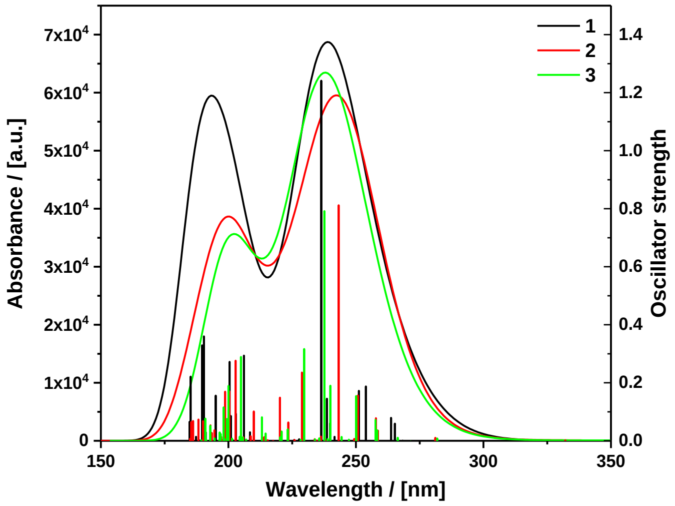

Figure S29. Theoretical absorption spectra of **1**, **2** and **3** (TDA-DFT rCAM-B3LYP/6-31G(d)) along with the oscillator strengths.

These subtle differences in the CT character can be followed in the natural transition orbital (NTO) representations (Figure S30). $S_0 \rightarrow S_1$ NTOs of **1-3** reflect the n-π* HOMO-LUMO orbital distribution and feature well-separated hole and particle with a certain overlap. The $S_0 \rightarrow S_{2,3}$ excitations of **1** involve

inter-acceptor LE transitions, while the folded skeleton of **2** and **3** is responsible for the hybrid LE-CT nature of $S_0 \rightarrow S_2$. Finally, the $S_0 \rightarrow S_3$ excitation of **3** displays an LE acceptor-localized character due to the presence of an additional nitrogen atom. Spirofluorene-localized T_1 NTOs are of LE nature for the three molecules. T_2 of **1** and **2**, and T_3 of **3** are electron-acceptor localized. While T_3 of compound **1** was found to rest on the acridine donor, T_3 of **2** and T_2 of **3** feature CT characters of hole-particle distribution.

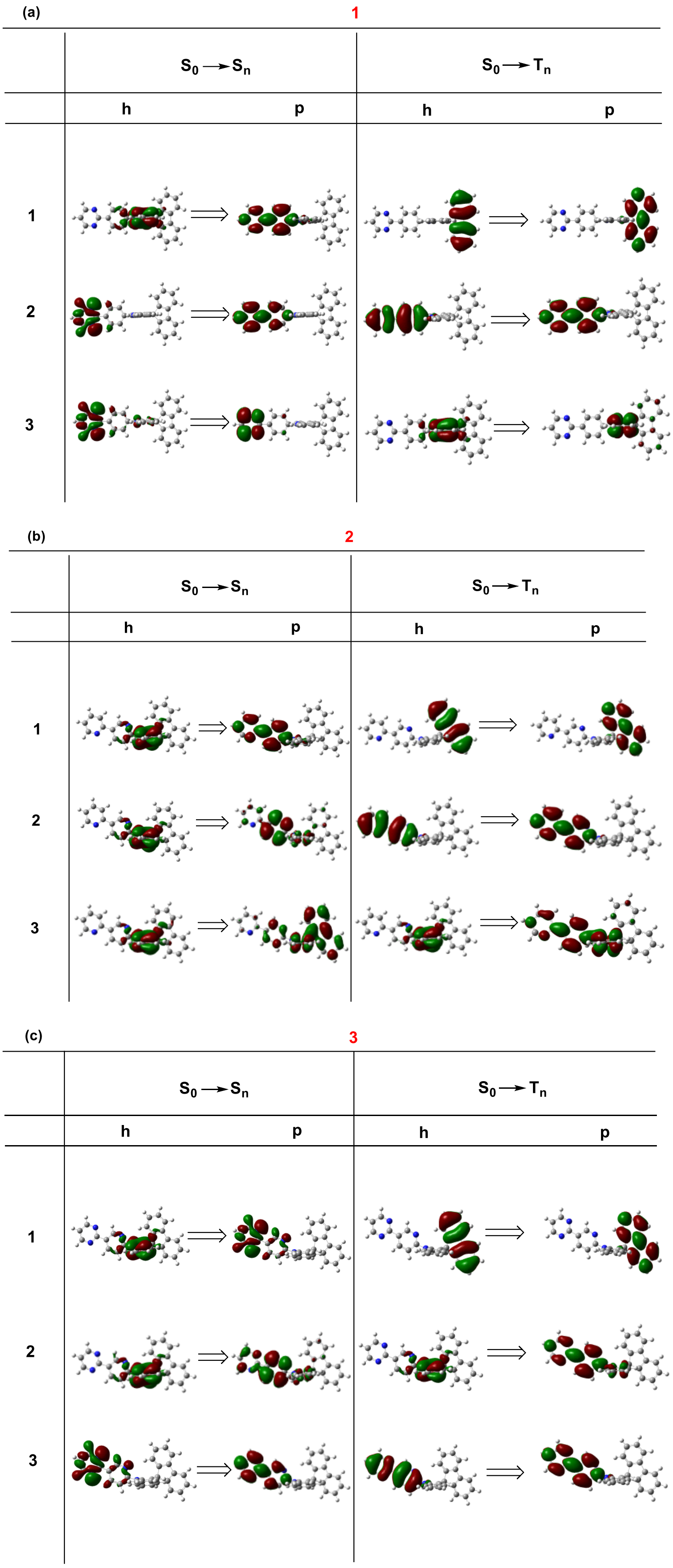

Figure S30. Singlet and triplet natural transition orbitals (NTO) of (a) **1**, (b) **2** and (c) **3** (TDA-DFT CAM-B3LYP/6-31G(d)).

In the case of the three derivatives the calculated S_1-T_1 energy gap (Figure S31) is relatively small (11-38 meV). The ΔE_{ST} values decrease in the following row: **1** (0.38 eV) > **2** (0.27 eV) > **3** (0.11 eV). Such progression aligns well with the increased electron accepting ability of the chromophores (see LUMO values, Figure S28), and the CT character trends, supported by the UV-Vis absorption spectra (Figure S26, S30).

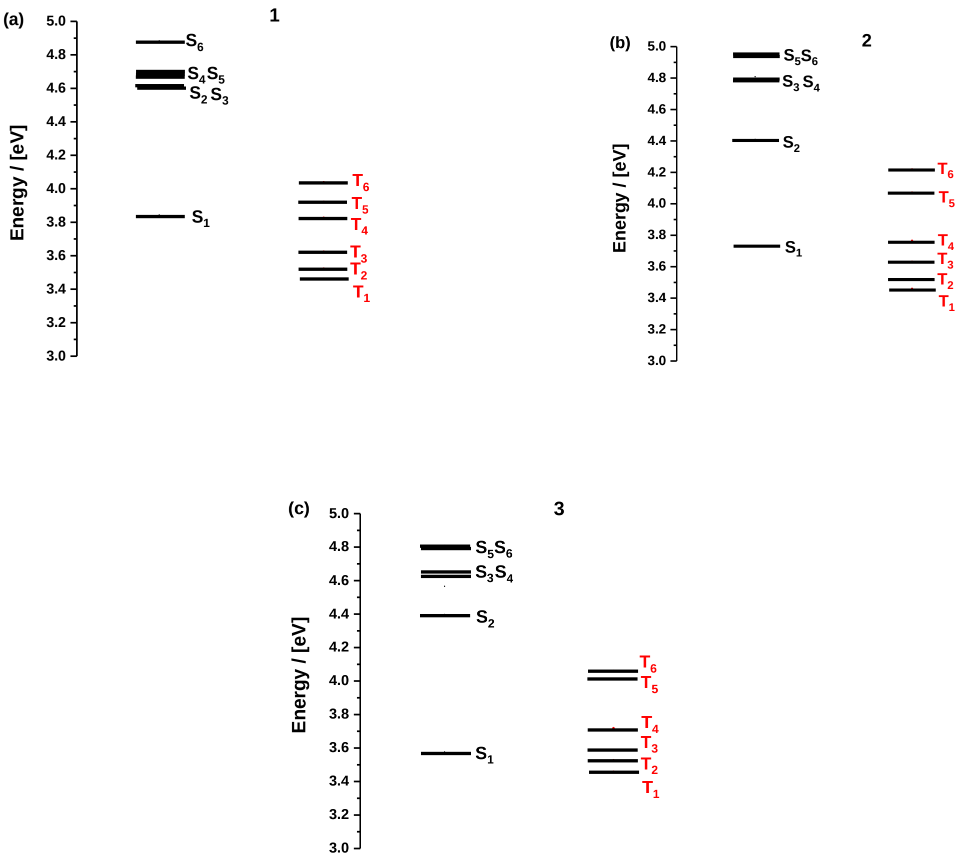

Figure S31. Singlet and triplet energy diagram of (a) **1**, (b) **2** and (c) **3** (TDA-DFT rCAM-B3LYP/6-31G(d).

In order to evaluate the probability of the rotations around the D-A bond in the S_1 state, total energy scans were accessed (Figure 3d). Firstly, there is no change in the equilibrium D-A angle. If H-bonding were active, we would expect the attractive interaction between donor H and acceptor N to influence the D-A angle towards planarization. No such change is observed. Curiously, the rotational barrier of **1** with a phenylene spacer, is higher than those of **2** and **3**, bearing the pyridinyl spacer. If there was rigidification in **2** or **3** due to H-bonding, the opposite trend would be expected. In order to reveal the origin of the differences in the rotational barriers, geometrical changes at different twists of the D-A angle were examined in detail (Figure S32). Remember that the S_1 state geometry of compound **1** has a

close to planar acridine unit, while those of **2** and **3** have acridines pre-folded by 19°. Upon the chromophore rotation around the D-A N-C bond, the planar acridine of **1** gradually folds to the value of 31° (D-A twist by 60°); so do the already pre-folded acridines of **2** and **3** (the value of 31° is reached at a 60° D-A twist). For compound **1**, the energetic barrier between the conformers with planar and folded acridine unit was found to be as small as 0.012 eV. To quantify the driving force behind the gradual acridine folding, the changes in the angle between the acridine and spirofluorene planes were examined (namely, the angle between C4-C1-N20, Figure S32). While in case of the pre-folded acridine in **2** and **3** this angle experiences only minor changes upon the rotation around the D-A bond (from 94.7° to 90.4°); a drastic drop from 117° to 107° is required to fold the acridine unit by 13° in **1** upon the D-A rotation by 10° (energy barrier 1.4 meV). Further rotation leads to further acridine folding to 30° and the value of 85.8° of the C4-C1-N20 angle. The energy loss essential to pre-fold the acridine in compound **1** translates eventually in the 0.27 eV barrier at the 60° D-A twist. As the acridine fragments are already pre-folded in **2** and **3**, the overall rotational energy barrier is 0.09 eV smaller for these compounds at a 60° D-A twist.

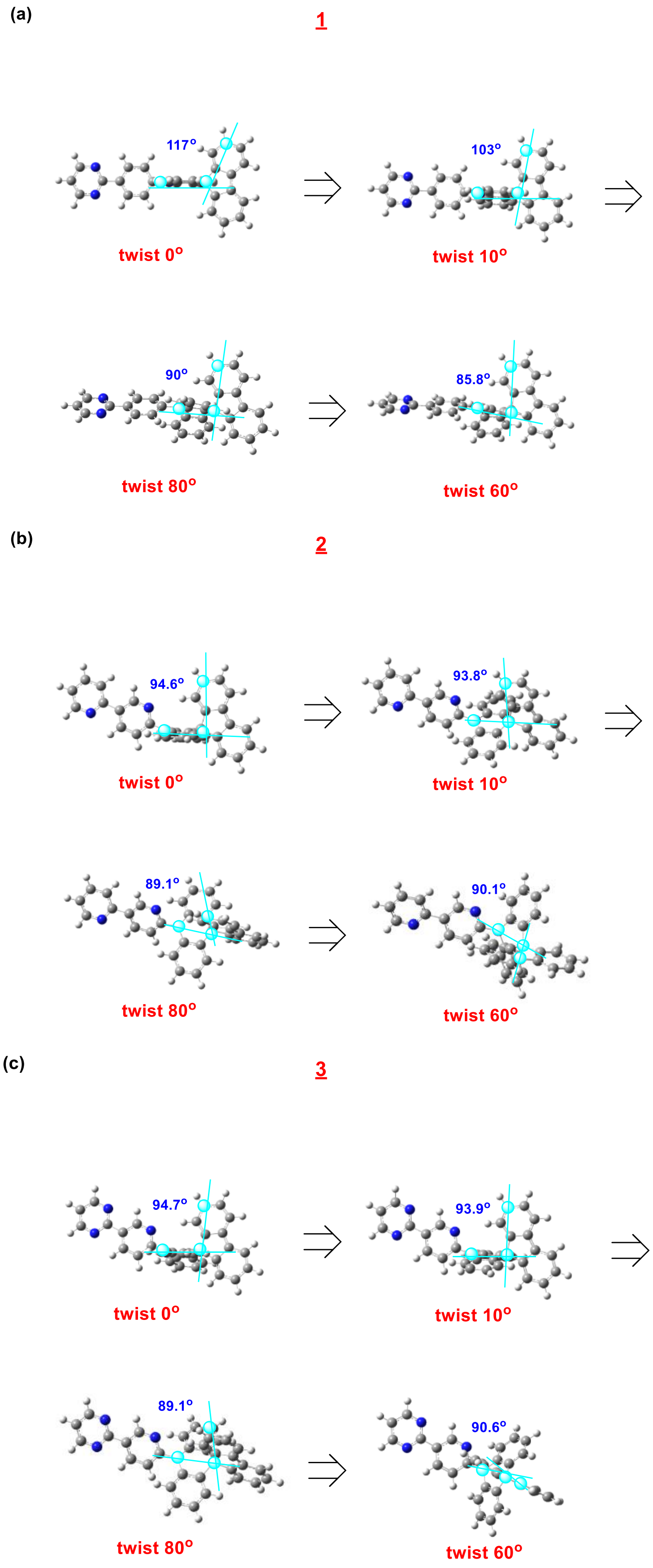

Figure S32. Conformational changes of (a) **1**, (b) **2** and (c) **3** (CAM-B3LYP/6-31G(d) at S_1 geometry. The angles between the atoms C4-C1-N20 are highlighted.

We speculate that the higher rotational barrier of **1** can be attributed to the steric hindrance due to the clashing of the *ortho*-hydrogens of the acridine and phenylene ring, hence ensuring the orthogonality.[33] On the contrary, this steric repulsion is minimized in **2** and **3** upon the introduction of the *ortho*-nitrogen atom, allowing freer rotation but without influencing the minimum angle of the potential energy surface. Furthermore, introduction of the *ortho*-nitrogen in **2** and **3** serves as the driving force behind the acridine-folding in these compounds, as witnessed by the electrostatic interaction between the nitrogen of the pyridine unit and the *ortho*-hydrogen of spiro-fluorene (2.85 Å) (Figure 1). While the nitrogen has a slightly larger atomic radius than the carbon, the absence of the hydrogen facilitates the rotation around the C-N bond, as opposed to commonly suggested H-bonding and planarized geometry. Acridine folding pushes the hydrogens on the donor even further away from the acceptor's plane, facilitating the rotation further. As a result, slightly higher conformational heterogeneity is expected for **2** and **3**.

In order to prove the validity of our computational results, ground and excited state geometries along with total energy scans were accessed with BMK and PBE0 functionals (Figure S33, Figure S34). While BMK has predicted planar acridines in case of the three compounds in ground and excited states, the trend in the rotational barriers fully supports the CAM-B3LYP results. Similarly to CAM-B3LYP, PBE0 predicted a folded acridine skeleton (16-22°) and a tilted electron acceptor fragment (29°) for **2** and **3** in both S_0 and S_1 states, as well as an analogues rotational barrier pattern. Potential energy surfaces for S_1 states support the same trend in the rotational barriers as the BMK functional (Figure S33, Figure S34), with compound **1** showing the highest energy barrier. Similarly to our previous results, we suggest that species with both folded and planar acridine can exist in solution owing to the negligible energy barrier.[30] As a result, we can conclude that no intramolecular H-bonding exists in **2** and **3**. Additionally, we hypothesize that H-bonding may not play as significant role as reported in other similar systems (where H-bonding is often assumed). The differences observed in optical and photophysical properties could arise instead from electronic and steric effects induced by the heteroatom-containing substituent.

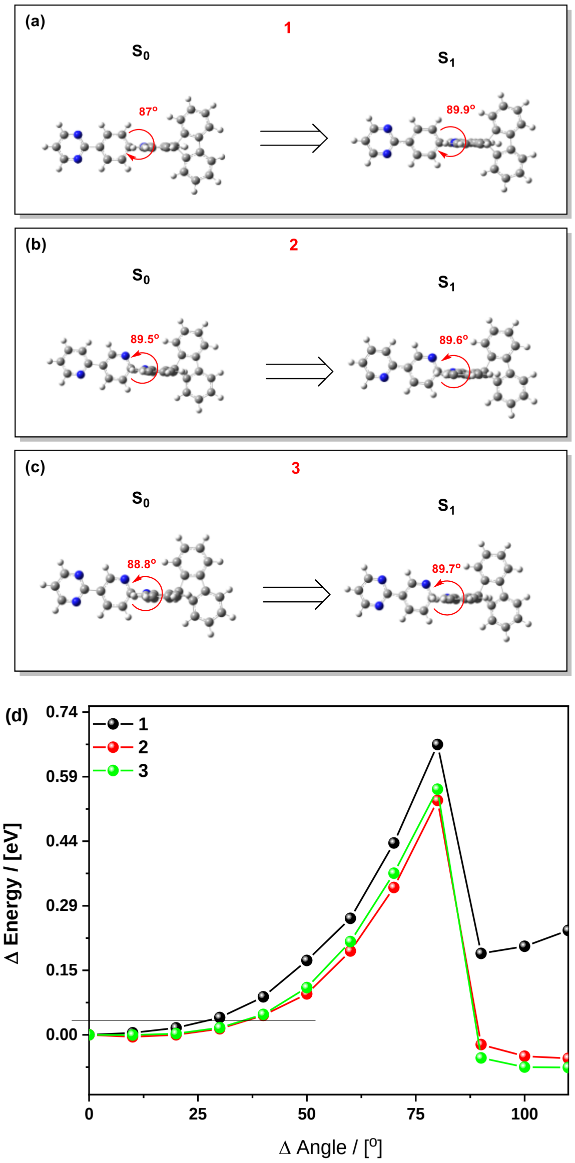

Figure S33. (a-c) Optimized ground state (rBMK/6-31G(d)) and excited state (TDA-DFT BMK/6-31G(d)) structures of **1**, **2** and **3**; (d) total energy scans of **1**, **2** and **3** (rBMK/6-31G(d)), calculated for the S_1 geometry.

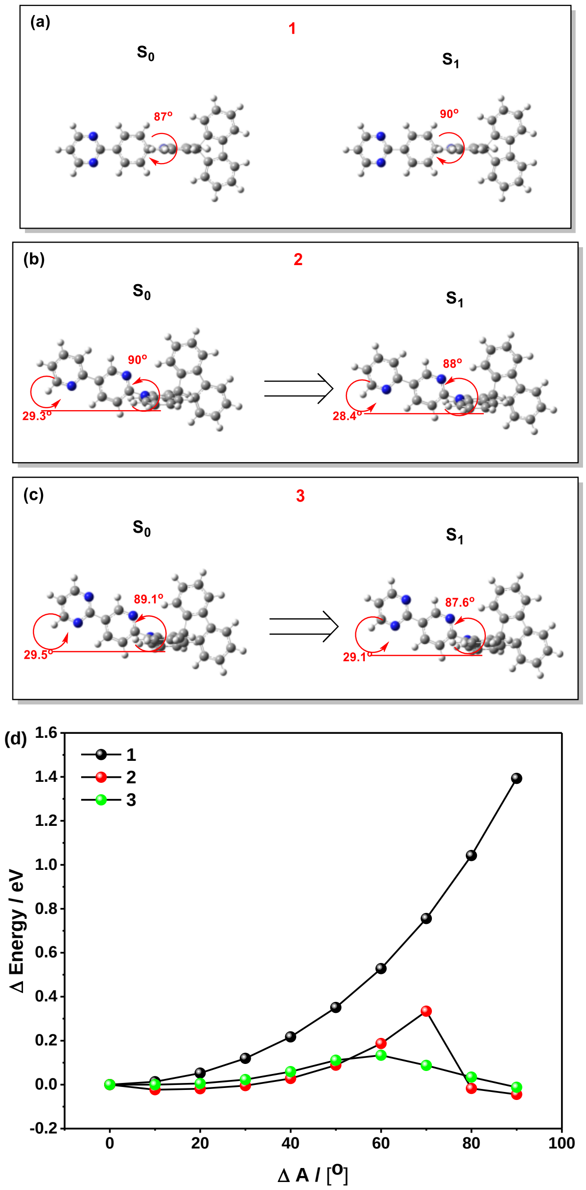

Figure S34. (a-c) Optimized ground state (rPBE0/6-31G(d)) and excited state (TDA-DFT PBE0/6-31G(d)) structures of **1**, **2** and **3**; (d) total energy scans of **1**, **2** and **3** (rPBE0/6-31G(d)), calculated for the S_1 geometry.

9. Cyclic Voltammetry

A trend in the electronic conjugation can be observed in the electronic properties of the three molecules, which were investigated by cyclic voltammetry of DMF solutions. Due to the usual perpendicular orientation of the donor and acceptor segments in TADF emitters, generally, the electronic properties of the full D-A structure can be derived from the respective behavior of the electronically decoupled donor and acceptor segments. While the electron affinity of the three materials cannot directly be compared due to the different acceptor units, all compounds **1-3** contain the same acridine donor segment, which, in principle, should allow for a direct comparison. Here, compounds **2** and **3** show elevated ionization potentials (IP) with respect to the parent structure **1**, potentially indicating a reduced (intrasegmental) electronic conjugation along the acridine donor system (i.e. the acridine plane), resulting from a more folded acridine segment of **2** and **3**. These results lead to the possible conclusion that the pronounced folding of the acridine segment does not result from packing effects within the crystal unit cells, but is an apparent feature of the ground state structure even in diluted solutions of the materials.

An alternative explanation of the lower ionization potential in **1** would be a scenario in which the interchanging of the bridging phenyl unit in **1** for the pyridinyl segments in **2** and **3** would result in an alleviated steric hindrance, leading to lower intersegmental dihedral angles in **2** and **3** and enhanced electronic conjugation, respectively. Due to the low impact on the electronic system when comparing the three compounds, a full planarization of the overall structures in **2** and **3** due to intramolecular H-bonding seems rather implausible.

Table S3. Overview of the electrochemical analysis of compounds **1-3** (performed in DMF (abs.), 0.1 M $TBAPF_6$, HOMO = -(IP+5.1) eV, LUMO = HOMO + Eg).

	IP (vs. Fc/Fc$^+$) [V]	EA (vs. Fc/Fc$^+$) [V]	Eg (V)	HOMO [eV]	LUMO [eV]
**1**	0.55	-2.36	2.91	-5.65	-2.74
**2**	0.61	-2.39	3.01	-5.71	-2.71
**3**	0.63	-2.20	2.83	-5.73	-2.90

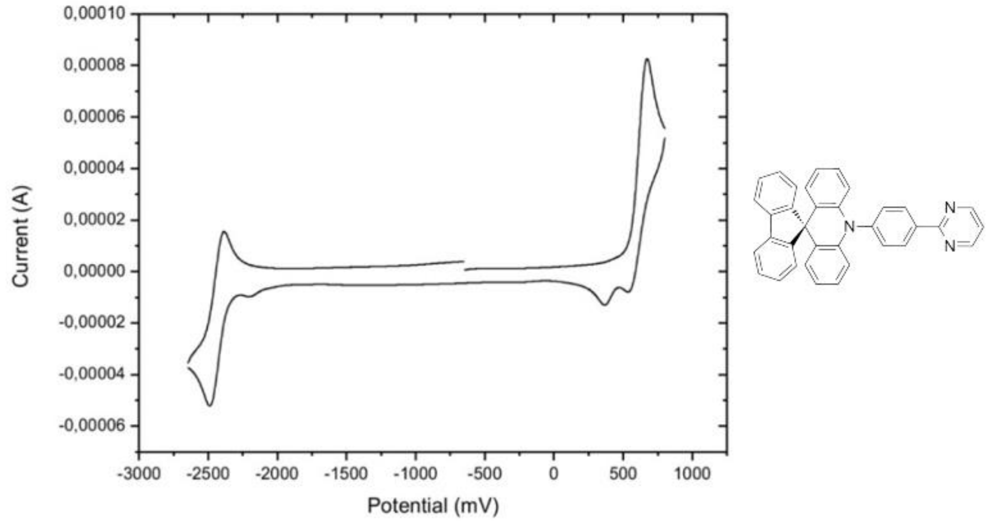

Figure S35. Cyclic voltammetry of compound **1** (DMF (abs.), 0.1 M $TBAPF_6$, corrected vs. Fc/Fc^+).

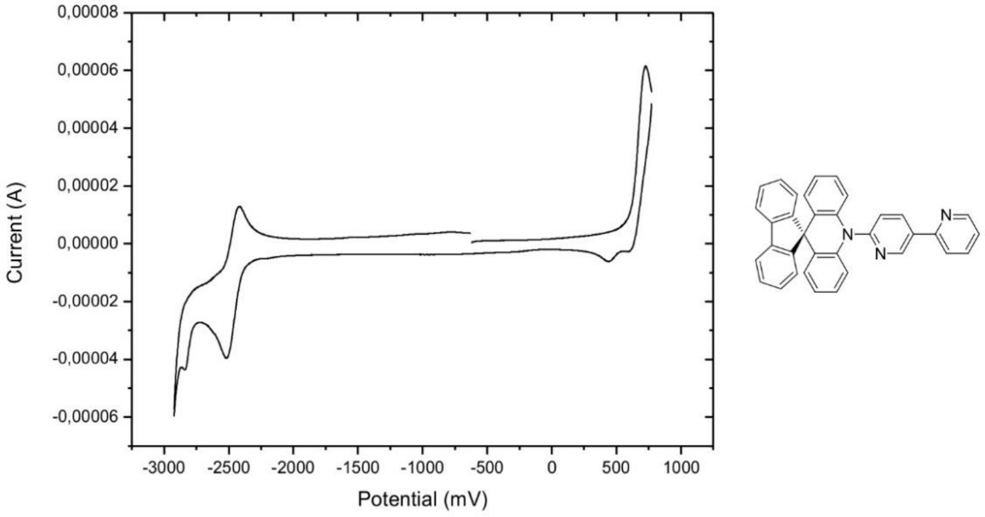

Figure S36. Cyclic voltammetry of compound **2** (DMF (abs.), 0.1 M $TBAPF_6$, corrected vs. Fc/Fc^+).

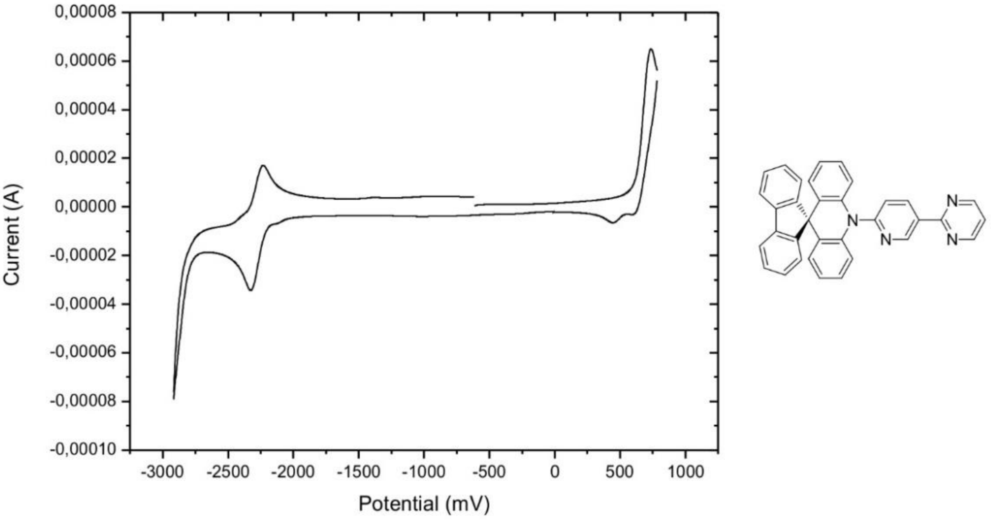

Figure S37. Cyclic voltammetry of compound **3** (DMF (abs.), 0.1 M $TBAPF_6$, corrected vs. Fc/Fc^+).

10. References

(1) Fulmer, G. R.; Miller, A. J. M.; Sherden, N. H.; Gottlieb, H. E.; Nudelman, A.; Stoltz, B. M.; Bercaw, J. E.; Goldberg, K. I., NMR Chemical Shifts of Trace Impurities: Common Laboratory Solvents, Organics, and Gases in Deuterated Solvents Relevant to the Organometallic Chemist. *Organometallics* **2010**, *29*, (9), 2176–2179.

(2) Krause, L.; Herbst-Irmer, R.; Sheldrick, G. M.; Stalke, D., Comparison of silver and molybdenum microfocus X-ray sources for single-crystal structure determination. *J. Appl. Crystallogr.* **2015**, *48*, (1), 3–10.

(3) Sheldrick, G. M., SHELXT - integrated space-group and crystal-structure determination. *Acta Crystallogr. A* **2015**, *71*, (Pt 1), 3–8.

(4) Sheldrick, G., Crystal structure refinement with SHELXL. *Acta Crystallogr. C* **2015**, *71*, (1), 3–8.

(5) Dolomanov, O. V.; Bourhis, L. J.; Gildea, R. J.; Howard, J. A. K.; Puschmann, H., OLEX2: a complete structure solution, refinement and analysis program. *J. Appl. Crystallogr.* **2009**, *42*, (2), 339–341.

(6) Park, H.-J.; Han, S. H.; Lee, J. Y.; Han, H.; Kim, E.-G., Managing Orientation of Nitrogens in Bipyrimidine-Based Thermally Activated Delayed Fluorescent Emitters To Suppress Nonradiative Mechanisms. *Chem. Mater.* **2018**, *30*, (10), 3215–3222.

(7) Pandidurai, J.; Jayakumar, J.; Senthilkumar, N.; Cheng, C.-H., Effects of intramolecular hydrogen bonding on the conformation and luminescence properties of dibenzoylpyridine-based thermally activated delayed fluorescence materials. *J. Mater. Chem. C* **2019**, *7*, (42), 13104–13110.

(8) Zhang, Q.; Sun, S.; Lv, X.; Liu, W.; Zeng, H.; Guo, R.; Ye, S.; Leng, P.; Xiang, S.; Wang, L., Manipulating the positions of CH···N in acceptors of pyrimidine–pyridine hybrids for highly efficient sky-blue thermally activated delayed fluorescent OLEDs. *Mater. Chem. Front.* **2018**, *2*, (11), 2054–2062.

(9) Oh, C. S.; Lee, H. L.; Hong, W. P.; Lee, J. Y., Benzothienopyrimidine as a co-planar type rigid acceptor for high external quantum efficiency in thermally activated delayed fluorescence emitters. *J. Mater. Chem. C* **2019**, *7*, (25), 7643–7653.

(10) Thangaraji, V.; Rajamalli, P.; Jayakumar, J.; Huang, M.-J.; Chen, Y.-W.; Cheng, C.-H., Quinolinylmethanone-Based Thermally Activated Delayed Fluorescence Emitters and the Application in OLEDs: Effect of Intramolecular H-Bonding. *ACS Appl. Mater. Interfaces* **2019**, *11*, (19), 17128–17133.

(11) Rajamalli, P.; Chen, D.; Li, W.; Samuel, I. D. W.; Cordes, D. B.; Slawin, A. M. Z.; Zysman-Colman, E., Enhanced thermally activated delayed fluorescence through bridge modification in sulfone-based emitters employed in deep blue organic light-emitting diodes. *J. Mater. Chem. C* **2019**, *7*, (22), 6664–6671.

(12) Dos Santos, P. L.; Chen, D.; Rajamalli, P.; Matulaitis, T.; Cordes, D. B.; Slawin, A. M. Z.; Jacquemin, D.; Zysman-Colman, E.; Samuel, I. D. W., Use of Pyrimidine and Pyrazine Bridges as a Design Strategy To Improve the Performance of Thermally Activated Delayed Fluorescence Organic Light Emitting Diodes. *ACS Appl Mater Interfaces* **2019**, *11*, (48), 45171–45179.

(13) Ma, F.; Zhao, G.; Zheng, Y.; He, F.; Hasrat, K.; Qi, Z., Molecular Engineering of Thermally Activated Delayed Fluorescence Emitters with Aggregation-Induced Emission via Introducing Intramolecular Hydrogen-Bonding Interactions for Efficient Solution-Processed Nondoped OLEDs. *ACS Appl. Mater. Interfaces* **2020**, *12*, (1), 1179–1189.

(14) Ma, F.; Cheng, Y.; Zhang, X.; Gu, X.; Zheng, Y.; Hasrat, K.; Qi, Z., Enhancing performance for blue TADF emitters by introducing intramolecular CH···N hydrogen bonding between donor and acceptor. *Dyes Pigm.* **2019**, *166*, 245–253.

(15) Rajamalli, P.; Senthilkumar, N.; Huang, P. Y.; Ren-Wu, C. C.; Lin, H. W.; Cheng, C. H., New Molecular Design Concurrently Providing Superior Pure Blue, Thermally Activated Delayed Fluorescence and Optical Out-Coupling Efficiencies. *J. Am. Chem. Soc.* **2017**, *139*, (32), 10948–10951.

(16) Ma, M.; Li, J.; Liu, D.; Mei, Y.; Dong, R., Rational Utilization of Intramolecular Hydrogen Bonds to Achieve Blue TADF with EQEs of Nearly 30% and Single Emissive Layer All-TADF WOLED. *ACS Appl. Mater. Interfaces* **2021**, *13*, (37), 44615–44627.

(17) Wang, L.; Cai, X.; Li, B.; Li, M.; Wang, Z.; Gan, L.; Qiao, Z.; Xie, W.; Liang, Q.; Zheng, N.; Liu, K.; Su, S.-J., Achieving Enhanced Thermally Activated Delayed Fluorescence Rates and Shortened Exciton Lifetimes by Constructing Intramolecular Hydrogen Bonding Channels. *ACS Appl. Mater. Interfaces* **2019**, *11*, (49), 45999–46007.

(18) Xu, J.; Wu, X.; Guo, J.; Zhao, Z.; Tang, B. Z., Sky-blue delayed fluorescence molecules based on pyridine-substituted acridone for efficient organic light-emitting diodes. *J. Mater. Chem. C* **2021**, *9*, 15505–15510.

(19) Xie, F.-M.; Zeng, X.-Y.; Zhou, J.-X.; An, Z.-D.; Wang, W.; Li, Y.-Q.; Zhang, X.-H.; Tang, J.-X., Intramolecular H-bond design for efficient orange–red thermally activated delayed fluorescence based on a rigid dibenzo[f,h]pyrido[2,3-b]quinoxaline acceptor. *J. Mater. Chem. C* **2020**, *8*, (44), 15728–15734.

(20) Chen, J.-X.; Xiao, Y.-F.; Wang, K.; Fan, X.-C.; Cao, C.; Chen, W.-C.; Zhang, X.; Shi, Y.-Z.; Yu, J.; Geng, F.-X.; Zhang, X.-H.; Lee, C.-S., Origin of thermally activated delayed fluorescence in a donor–acceptor type emitter with an optimized nearly planar geometry. *J. Mater. Chem. C* **2020**, *8*, (38), 13263–13269.

(21) Hempe, M.; Schnellbächer, L.; Wiesner, T.; Reggelin, M., meta- and para-Functionalized Thermally Crosslinkable OLED-Materials through Selective Transition-Metal-Catalyzed Cross-Coupling Reactions. *Synthesis* **2017**, *49*, (19), 4489–4499.

(22) Budén, M. E.; Vaillard, V. A.; Martin, S. E.; Rossi, R. A., Synthesis of Carbazoles by Intramolecular Arylation of Diarylamide Anions. *J. Org. Chem.* **2009**, *74*, (12), 4490–4498.

(23) Li, B.; Li, Z.; Hu, T.; Zhang, Y.; Wang, Y.; Yi, Y.; Guo, F.; Zhao, L., Highly efficient blue organic light-emitting diodes from pyrimidine-based thermally activated delayed fluorescence emitters. *J. Mater. Chem. C* **2018**, *6*, (9), 2351–2359.

(24) Neumann, T.; Benajiba, L.; Göring, S.; Stegmaier, K.; Schmidt, B., Evaluation of Improved Glycogen Synthase Kinase-3α Inhibitors in Models of Acute Myeloid Leukemia. *J. Med. Chem.* **2015**, *58*, (22), 8907–8919.

(25) Burzicki, G.; Voisin-Chiret, A. S.; Santos, J. S.-d. O.; Rault, S., Synthesis of New [2,3′:6′,3″]Terpyridines Using Iterative Cross-Coupling Reactions. *Synthesis* **2010**, (16), 2804–2810.

(26) Rowland, R. S.; Taylor, R., Intermolecular Nonbonded Contact Distances in Organic Crystal Structures: Comparison with Distances Expected from van der Waals Radii. *J. Phys. Chem.* **1996**, *100*, (18), 7384–7391.

(27) Wong, N.-B.; Cheung, Y.-S.; Wu, D. Y.; Ren, Y.; Wang, X.; Tian, A. M.; Li, W.-K., A theoretical study of the C–H···N hydrogen bond in the methane–ammonia complex. *J. Mol. Struct. Theochem* **2000**, *507*, (1), 153–156.

(28) Al-Saadon, R.; Sutton, C.; Yang, W., Accurate Treatment of Charge-Transfer Excitations and Thermally Activated Delayed Fluorescence Using the Particle–Particle Random Phase Approximation. *J. Chem. Theory Comput.* **2018**, *14*, (6), 3196–3204.

(29) Wex, B.; Kaafarani, B. R., Perspective on carbazole-based organic compounds as emitters and hosts in TADF applications. *J. Mater. Chem. C* **2017**, *5*, (34), 8622–8653.

(30) Hempe, M.; Kukhta, N. A.; Danos, A.; Fox, M. A.; Batsanov, A. S.; Monkman, A. P.; Bryce, M. R., Vibrational Damping Reveals Vibronic Coupling in Thermally Activated Delayed Fluorescence Materials. *Chem. Mater.* **2021**, *33*, (9), 3066–3080.

(31) Kukhta, N. A.; Higginbotham, H. F.; Matulaitis, T.; Danos, A.; Bismillah, A. N.; Haase, N.; Etherington, M. K.; Yufit, D. S.; McGonigal, P. R.; Gražulevičius, J. V.; Monkman, A. P., Revealing resonance effects and intramolecular dipole interactions in the positional isomers of benzonitrile-core thermally activated delayed fluorescence materials. *J. Mater. Chem. C* **2019**, *7*, (30), 9184–9194.

(32) Metri, N.; Sallenave, X.; Plesse, C.; Beouch, L.; Aubert, P.-H.; Goubard, F.; Chevrot, C.; Sini, G., Processable Star-Shaped Molecules with Triphenylamine Core as Hole-Transporting Materials: Experimental and Theoretical Approach. *J. Phys. Chem. C* **2012**, *116*, (5), 3765–3772.

(33) Salah, L.; Etherington, M. K.; Shuaib, A.; Danos, A.; Nazeer, A. A.; Ghazal, B.; Prlj, A.; Turley, A. T.; Mallick, A.; McGonigal, P. R.; Curchod, B. F. E.; Monkman, A. P.; Makhseed, S., Suppressing dimer formation by increasing conformational freedom in multi-carbazole thermally activated delayed fluorescence emitters. *J. Mater. Chem. C* **2021**, *9*, (1), 189–198.
